# Protective effect of PDE4B subtype-specific inhibition in an *App* knock-in mouse model for Alzheimer’s disease

**DOI:** 10.1038/s41386-024-01852-z

**Published:** 2024-03-23

**Authors:** Paul Armstrong, Hüseyin Güngör, Pariya Anongjanya, Clare Tweedy, Edward Parkin, Jamie Johnston, Ian M. Carr, Neil Dawson, Steven J. Clapcote

**Affiliations:** 1 School of Biomedical Sciences, University of Leeds, LS2 9JT Leeds, UK; 2 Division of Biomedical and Life Sciences, Faculty of Health and Medicine, Lancaster University, LA1 4YG Lancaster, UK; 3 Department of Veterinary Pharmacology and Toxicology, Faculty of Veterinary Medicine, Cumhuriyet University, Sivas, 58140 Turkey; 4 Leeds Institute of Medical Research, University of Leeds, LS9 7TF Leeds, UK

**Keywords:** Alzheimer's disease, Long-term memory

## Abstract

Meta-analysis of genome-wide association study data has implicated *PDE4B* in the pathogenesis of Alzheimer’s disease (AD), the leading cause of senile dementia. *PDE4B* encodes one of four subtypes of cyclic adenosine monophosphate (cAMP)-specific phosphodiesterase-4 (PDE4A–D). To interrogate the involvement of PDE4B in the manifestation of AD-related phenotypes, the effects of a hypomorphic mutation (*Pde4b*^Y358C^) that decreases PDE4B’s cAMP hydrolytic activity were evaluated in the *App*^NL-G-F^ knock-in mouse model of AD using the Barnes maze test of spatial memory, ^14^C-2-deoxyglucose autoradiography, thioflavin-S staining of β-amyloid (Aβ) plaques, and inflammatory marker assay and transcriptomic analysis (RNA sequencing) of cerebral cortical tissue. At 12 months of age, *App*^NL-G-F^ mice exhibited spatial memory and brain metabolism deficits, which were prevented by the hypomorphic PDE4B in *App*^NL-G-F^/*Pde4b*^Y358C^ mice, without a decrease in Aβ plaque burden. RNA sequencing revealed that, among the 531 transcripts differentially expressed in *App*^NL-G-F^ versus wild-type mice, only 13 transcripts from four genes – *Ide*, *Btaf1*, *Padi2,* and *C1qb* – were differentially expressed in *App*^NL-G-F^/*Pde4b*^Y358C^ versus *App*^NL-G-F^ mice, identifying their potential involvement in the protective effect of hypomorphic PDE4B. Our data demonstrate that spatial memory and cerebral glucose metabolism deficits exhibited by 12-month-old *App*^NL-G-F^ mice are prevented by targeted inhibition of PDE4B. To our knowledge, this is the first demonstration of a protective effect of PDE4B subtype-specific inhibition in a preclinical model of AD. It thus identifies PDE4B as a key regulator of disease manifestation in the *App*^NL-G-F^ model and a promising therapeutic target for AD.

## Introduction

Alzheimer’s disease (AD) is the leading cause of dementia and disability in old age, with pathological features including extracellular plaque deposits of the β-amyloid (Aβ) peptide and an estimated heritability of ∼60% [1–3]. Although recent clinical trials of Aβ-targeting monoclonal antibodies resulted in moderately less cognitive decline in people with early AD [4, 5], no therapeutics capable of halting or reversing the progression of the disease have been described.

The risk of developing senile dementia is 34% higher in individuals with gastroesophageal reflux disease (GERD) compared with controls [6]. Meta-analysis of genome-wide association study (GWAS) data indicates that AD and GERD share seven genome-wide significant susceptibility loci [7]. Among the implicated genes, *PDE4B*, encoding one of four subtypes of cyclic adenosine monophosphate (cAMP)-specific phosphodiesterase-4 (PDE4A–D), was proposed as a plausible therapeutic target that should be investigated further [7].

Across various tissues, PDE4B is expressed in five isoforms (PDE4B1–5) [8]. In adult mammalian brain tissue, *Pde4b* transcripts have been identified by single-cell RNA sequencing in nearly all subclasses of GABAergic (gamma-aminobutyric acidergic) inhibitory neurons and glutamatergic excitatory neurons, and in some types of glial cell (Figs. S1 and S2) [9, 10]. PDE4B transcription was shown to be upregulated in primary rat microglial cell cultures by exposure to Aβ peptides, resulting in induction of the inflammatory cytokine TNFα, which was markedly decreased by the non-subtype-selective (pan-) PDE4 inhibitor rolipram (targeting all of four subtypes, PDE4A–D) [11].

The super-family of phosphodiesterases (PDE1–11) has long been considered as potential targets for AD therapy [12–19]. Transgenic amyloid precursor protein (APP)-overexpressing mouse models of AD have shown amelioration of cognitive deficits following treatment with the pan-PDE4 inhibitors rolipram [20–22], roflumilast [23, 24] and FFPM [25], and with the PDE4D subtype-selective inhibitors GEBR-7b [26] and GEBR-32a [27]. Rolipram had no effect on Aβ peptide levels or plaque load in the PS/APP and Tg2576 strains [20, 21], although treatment over 24 days was shown to decrease Aβ peptide levels in brain tissue from 11-month-old 3xTg-AD mice [22]. Prior to the genetic validation of PDE4B by the GWAS meta-analysis [7], no work had been published in this context specifically on the PDE4B subtype.

We previously showed that a hypomorphic mutation (*Pde4b*^Y358C^), which affects all PDE4B isoforms and decreases the enzyme’s cAMP hydrolytic activity by 27%, results in increased phosphorylation of CREB (cAMP response element binding protein) and cognitive enhancement in young adult (12-week-old) C57BL/6J mice [28]. To interrogate the involvement of PDE4B in the manifestation of AD-related phenotypes, we evaluated the neurocognitive effects of the *Pde4b*^Y358C^ hypomorph in the *App*^NL-G-F^ knock-in mouse model [29], which shows Aβ peptide accumulation, neuroinflammation, and cognitive impairment in an age-dependent manner, without the non-physiological overexpression of APP [30].

The murine *App*^NL-G-F^ allele has a humanized Aβ peptide sequence, owing to three amino acid substitutions (G676R, F681Y, R684H; exon 16), and harbors three familial AD mutations: Swedish (K670*N*, M671*L*; exon 16), Arctic (E693*G*; exon 17), and Iberian (I716*F*; exon 17) [29]. The lecanemab monoclonal antibody, developed as an immunotherapy and tested in recent successful clinical trials, was raised against the Arctic Aβ variant [4].

Herein, we report that spatial memory and brain metabolism deficits exhibited by 12-month-old homozygous *App*^NL-G-F^ mice are counteracted by the hypomorphic PDE4B, without decreasing Aβ plaque burden. This genetically mediated protective effect identifies PDE4B as a key regulator of disease manifestation in the *App*^NL-G-F^ model and a promising therapeutic target for AD.

## Methods and materials

For more detailed methodology, see the Supplementary Materials and Methods.

### Mice

The *Pde4b*^Y358C^ mutation from the B6.C-*Pde4b*^enu1H^ line [28] was bred into the C57BL/6-*App*^tm3.1(NL-G-F)Tcs^ (*App*^NL-G-F^) line [29] to generate *App*^+/+^;*Pde4b*^+/+^ (wild-type; WT), *App*^NL-G-F/NL-G-F^;*Pde4b*^+/+^ (*App*^NL-G-F^) and *App*^NL-G-F/NL-G-F^;*Pde4b*^Y358C/Y358C^ (*App*^NL-G-F^/*Pde4b*^Y358C^) littermates for phenotypic testing. The mouse experiments were conducted in accordance with the UK Animals (Scientific Procedures) Act 1986 under UK Home Office licences and approved by institutional Animal Welfare and Ethical Review Bodies at the University of Leeds and Lancaster University.

### Barnes maze

The Barnes maze test was conducted as described previously with slight modifications [31].

### ^14^C-2-DG autoradiography

The ^14^C-2-deoxyglucose (^14^C-2-DG) functional brain imaging technique was undertaken as described previously [32, 33].

### Inflammation assay

Cortical lysates (2.5 mg in 100 µl) were analyzed for 32 inflammatory markers in duplicate using the Mouse Cytokine/Chemokine 32-Plex Array (Eve Technologies).

### Thioflavin-S staining

Aβ deposits in 4% paraformaldehyde-fixed brain sections were stained with thioflavin-S (Toronto Research Chemicals).

### RNA sequencing

Sequence data from a NovaSeq 6000 (Illumina) instrument were processed using the R package DSeq2 [34] to identify differentially expressed transcripts with *p*-values < 0.01 adjusted (adj.) for multiple testing using the Benjamini-Hochberg false discovery rate method [35].

### Western blotting

Western blotting was undertaken as previously described [31], using rabbit polyclonal antibody PC730 to insulin-degrading enzyme (IDE) (Millipore) or mouse monoclonal antibody AC-15 to β-actin (Sigma-Aldrich).

### Statistical analysis

All data values in the text and figure legends are represented as the mean ± standard error of the mean. A statistically significant difference was set at *p* < 0.01 for RNA sequencing and *p* < 0.05 for other data.

## Results

### Protective effect of hypomorphic PDE4B on spatial memory in *App*^NL-G-F^ mice

In the Barnes maze, *App*^NL-G-F^ mice previously showed deficient spatial learning and intact spatial reference memory at 8 months of age [36]. Other studies report intact spatial learning but subtly deficient spatial reference memory in *App*^NL-G-F^ mice aged 4.5 months [37] and 6 months [38]. We therefore aged mice to 12 months before evaluating their abilities in the Barnes maze.

**Fig. 1:**
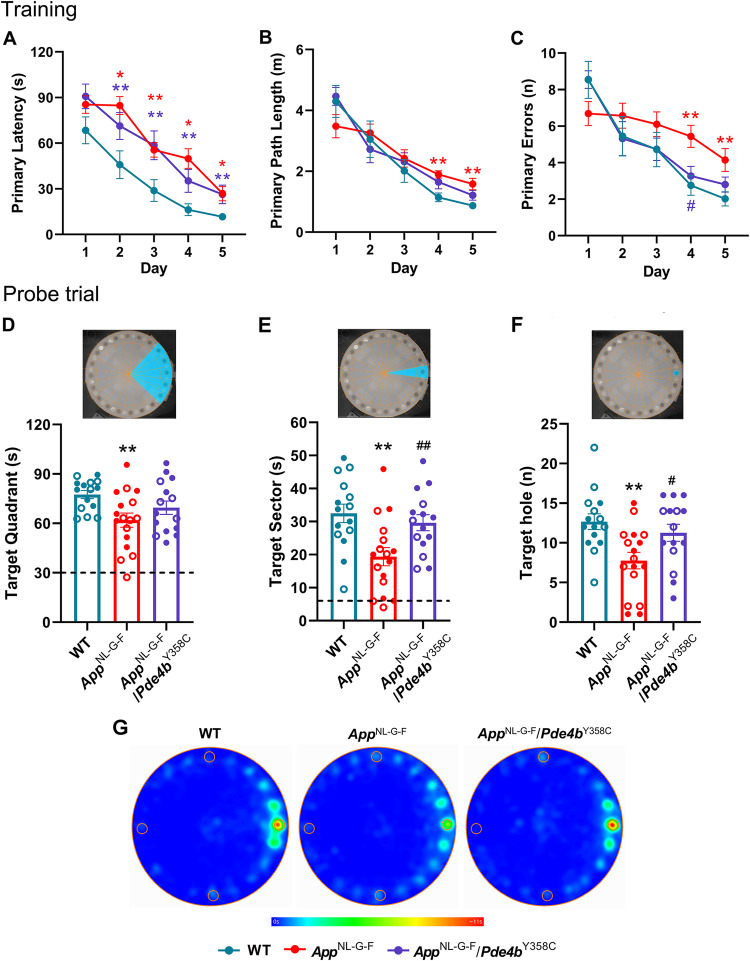
Protective effect of hypomorphic PDE4B on spatial memory in *App*^NL-G-F^ mice assessed by the Barnes maze. **A** Primary latency (s). All three genotypes showed a significant decrease over the five training days (Friedman’s ANOVA, WT: χ^2^_4_ = 37.819, *p* < 0.001; *App*^NL-G-F^: χ^2^_4_ = 39.847, *p* < 0.001; *App*^NL-G-F^/*Pde4b*^Y358C^: χ^2^_4_ = 43.518, *p* < 0.001). Genotypic differences were observed from day 2 (Kruskal-Wallis test, day 2: χ^2^_2_ = 8.939, *p* = 0.011; day 3: χ^22^ = 10.142, *p* = 0.006; day 4: χ^2^_2_ = 15.830, *p* < 0.001; day 5: χ^2^_2_ = 10.712, *p* = 0.005). **B** Primary path length (m). All three genotypes showed a significant decrease over the five training days (Friedman’s ANOVA, WT: χ^2^_4_ = 34.880, *p* < 0.001; *App*^NL-G-F^: χ^2^_4_ = 27.6^2^_4_, *p* < 0.001; *App*^NL-G-F^/*Pde4b*^Y358C^: χ^2^_4_ = 37.867, *p* < 0.001). Genotypic differences were observed on days 4 and 5 (Kruskal-Wallis test, day 4: χ^2^_2_ = 10.078, *p* = 0.006; day 5: χ^2^_2_ = 9.441, *p* = 0.009). **C** Number of errors. All three genotypes showed a significant decrease over the five training days (Friedman’s ANOVA, WT: χ^2^_4_ = 26.726, *p* < 0.001; *App*^NL-G-F^: χ^2^_4_ = 15.075, *p* = 0.005; *App*^NL-G-F^/*Pde4b*^Y358C^: χ^2^_4_ = 34.562, *p* < 0.001). Genotypic differences were observed on days 4 and 5 (Kruskal-Wallis test, day 4: χ^2^_2_ = 11.461, *p* = 0.003; day 5: χ^2^_2_ = 7.209, *p* = 0.027). **D** Time (s) spent in target quadrant. Genotypic differences were observed (ANOVA, *F*_2,41_ = 4.304, *p* = 0.02). **E** Time (s) spent in target sector. Genotypic differences were observed (ANOVA, *F*_2,41_ = 6.111, *p* = 0.005). **F** Number of head entries into the target hole annulus. Genotypic differences were observed (ANOVA, *F*_2,41_ = 5.547, *p* = 0.007). **G** Heat maps showing the cumulative time spent in localities of the arena. 12-month-old WT (*n* = 15), *App*^NL-G-F^ (*n* = 17) and *App*^NL-G-F^/*Pde4b*^Y358C^ (*n* = 15) mice. Data are plotted as mean ± SEM. *p < 0.05; **p < 0.01 vs. WT. ^#^*p* < 0.05; ^##^*p* < 0.01 vs. *App*^NL-G-F^. Open circles, females; closed circles, males; broken line, chance level.

Over 5 days of training, 12-month-old *App*^NL-G-F^ and *App*^NL-G-F^/*Pde4b*^Y358C^ mice took significantly longer to reach the target hole than WT controls (primary latency; Fig. 1A). *App*^NL-G-F^ mice also moved more slowly on days 1–3 (velocity; Fig. S3A), took a longer path to the target hole (primary path length; Fig. 1B), and made more errors (primary errors; Fig. 1C) compared with WT controls. During the probe trial, *App*^NL-G-F^ mice spent less time than WT mice in the target quadrant (25% of arena) (Fig. 1D), less time than WT and *App*^NL-G-F^/*Pde4b*^Y358C^ mice in the target sector (5% of arena) (Fig. 1E), and made fewer head entries than WT and *App*^NL-G-F^/*Pde4b*^Y358C^ mice into the target hole annulus (Fig. 1F). These measures were not different in *App*^NL-G-F^/*Pde4b*^Y358C^ mice relative to WT controls. Velocity was not significantly different between genotypes (Fig. S3C). *App*^NL-G-F^ mice thus displayed a spatial memory deficit that was prevented by the hypomorphic PDE4B present in *App*^NL-G-F^/*Pde4b*^Y358C^ mice.

### Protective effect of hypomorphic PDE4B on brain metabolism in *App*^NL-G-F^ mice

**Fig. 2:**
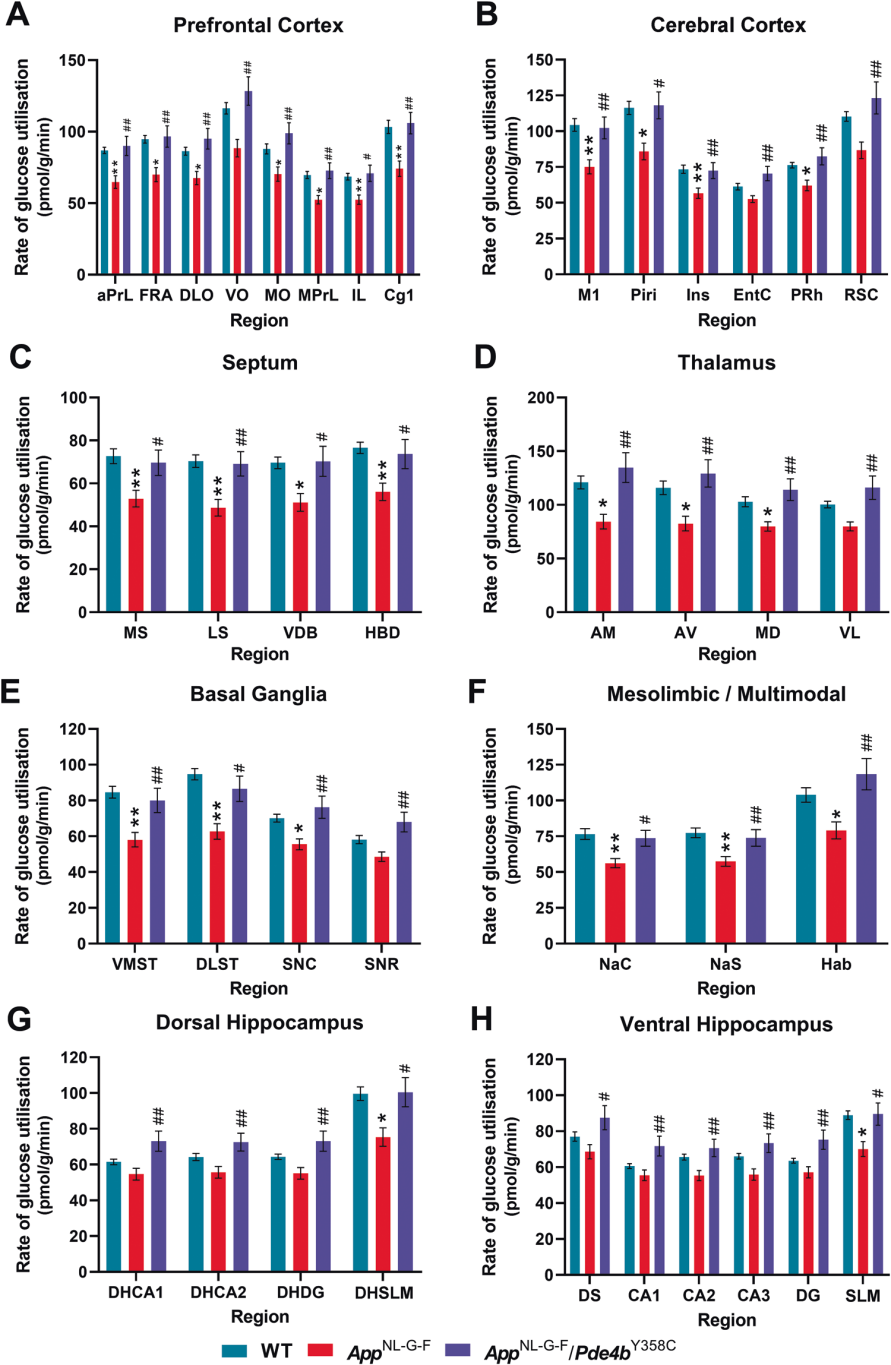
Protective effect of hypomorphic PDE4B on brain metabolism in *App*^NL-G-F^ mice assessed by ^14^C-2-DG brain imaging. *App*^NL-G-F^ mice show hypometabolism that is corrected in *App*^NL-G-F^/*Pde4b*^Y358C^ mice in multiple subfields of the PFC (**A**), cerebral cortex (**B**), septum (**C**), thalamus (**D**), basal ganglia (**E**), and mesolimbic pathway (**F**). *App*^NL-G-F^ also show hypometabolism selectively in the DHSLM and SLM that was corrected in *App*^NL-G-F^/*Pde4b*^Y358C^ mice (**G**, **H**). In other hippocampal subfields, *App*^NL-G-F^ mice did not show hypometabolism, whereas metabolism was enhanced in these subfields in *App*^NL-G-F^/*Pde4b*^Y358C^ mice. 13-month-old WT (*n* = 15), *App*^NL-G-F^ (*n* = 17) and *App*^NL-G-F^/*Pde4b*^Y358C^ (*n* = 15) mice. Data are plotted as mean ± SEM. Student’s *t-*test with Bonferonni-Holm post hoc correction for multiple comparisons. *p < 0.05; **p < 0.01 vs. WT. ^#^*p* < 0.05; ^##^*p* < 0.01 vs. *App*^NL-G-F^. Brain region abbreviations are defined in Table S1.

**Fig. 3:**
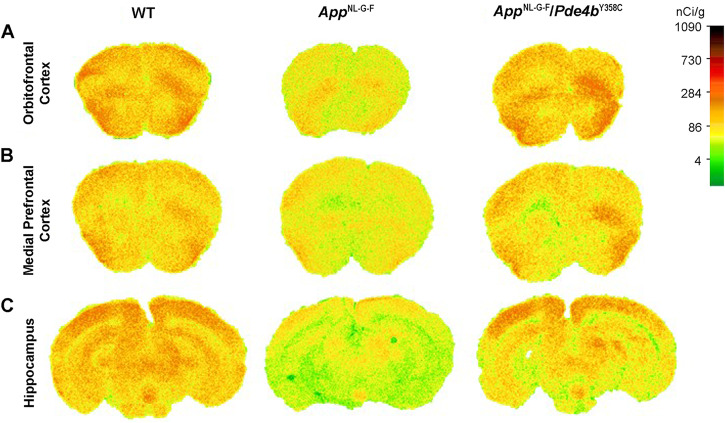
Exemplar ^14^C-2-DG color-coded autoradiographs obtained from coronal brain sections of 13-month-old WT, *App*^NL-G-F^, and *App*^NL-G-F^/*Pde4b*^Y358C^ mice. **A** Orbitofrontal cortex (+2.46 mm from bregma). **B** Medial PFC ( + 1.98 mm from bregma). **C** Hippocampus (−3.16 mm from bregma). Higher rates of metabolism are indicated by red/orange and lower rates indicated by yellow/white. Scale bar indicates tissue ^14^C concentration (nCi/g).

An increasing number of studies have shown that the onset and progression of AD are closely linked to glucose hypometabolism in the brain [39]. Hence, we assessed cerebral glucose metabolism as a function of neurogenic activity in the *App*^NL-G-F^ model using the translational ^14^C-2-DG brain imaging technique. In multiple brain regions, including the stratum lacunosum-moleculare (SLM) in hippocampal CA1 and subfields of the prefrontal cortex (PFC), glucose utilization was significantly lower in 13-month-old *App*^NL-G-F^ mice than in WT controls. However, significantly increased glucose utilization was observed in *App*^NL-G-F^/*Pde4b*^Y358C^ relative to *App*^NL-G-F^ mice in many of these regions, with cerebral metabolism being restored to the same level as that observed in WT controls (Fig. 2A–F). Aside from the SLM, glucose utilization in the hippocampus was not significantly different in *App*^NL-G-F^ and *App*^NL-G-F^/*Pde4b*^Y358C^ mice relative to WT controls, although a significant increase in hippocampal metabolism was observed in *App*^NL-G-F^/*Pde4b*^Y358C^ versus *App*^NL-G-F^ mice (Fig. 2G, H). Exemplar color-coded autoradiographs of coronal brain sections are shown in Fig. 3. Full data are shown in Tables S1 & S2. *App*^NL-G-F^ mice thus displayed a widespread cerebral hypometabolism that was prevented by the hypomorphic PDE4B present in *App*^NL-G-F^/*Pde4b*^Y358C^ mice.

Since Aβ deposition in cerebral gray matter is one of the hallmark pathologies of AD [1], we evaluated whether the Aβ plaque burden in 12-month-old *App*^NL-G-F^ mice was affected by hypomorphic PDE4B. The percentage of surface area occupied by thioflavin-S-stained Aβ plaques was not significantly different between *App*^NL-G-F^ and *App*^NL-G-F^/*Pde4b*^Y358C^ mice (Fig. S4).

### Partial attenuation of neuroinflammation in *App*^NL-G-F^ mice by hypomorphic PDE4B

**Fig. 4:**
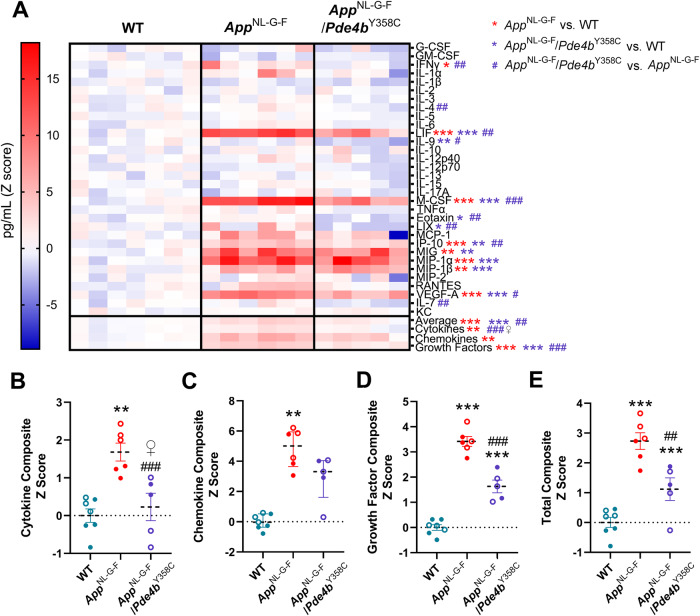
Partial attenuation of neuroinflammation in *App*^NL-G-F^ mice by hypomorphic PDE4B. **A** Heatmap analysis of Z scores of 32 inflammatory markers, each column indicating a different mouse. *App*^NL-G-F^ mice show elevated levels of inflammatory markers, which are partly decreased in *App*^NL-G-F^/*Pde4b*^Y358C^ mice. Composite Z scores for cytokines (**B**), chemokines (**C**), growth factors (**D**), and all 32 markers (total; **E**) are elevated in *App*^NL-G-F^ mice but modulated by the PDE4B inhibition in *App*^NL-G-F^/*Pde4b*^Y358C^ mice. 12-month-old WT (*n* = 7), *App*^NL-G-F^ (*n* = 6) and *App*^NL-G-F^/*Pde4b*^Y358C^ (*n* = 5) mice. Data are plotted as mean ± SEM. *p < 0.05; **p < 0.01; ***p < 0.001 vs. WT. ^#^*p* < 0.05; ^##^*p* < 0.01; ^###^*p* < 0.001 vs. *App*^NL-G-F^. Open circles, females; closed circles, males; ♀, significant difference in females only.

The involvement of neuroinflammation in AD is supported by accumulating evidence, including the manifestation of microgliosis in *App*^NL-G-F^ mice from 9 months of age [29, 40]. As PDE4B is the predominant negative modulator of cAMP signaling in microglia [41], which release elevated levels of inflammatory markers when chronically activated [42], we evaluated the brain levels of 32 inflammatory markers in 12-month-old mice. Brain lysates from *App*^NL-G-F^ mice showed increased levels of three cytokines (IFNγ, LIF & M-CSF; Fig. S5), four chemokines (IP-10, MIG, MIP-1α & MIP-1β), and one growth factor (VEGF-A; Fig. S6) compared with WT lysates (Fig. 4A). The levels of five of these markers (IFNγ, LIF, M-CSF, IP-10 & VEGF-A) were significantly decreased in *App*^NL-G-F^/*Pde4b*^Y358C^ compared with *App*^NL-G-F^ lysates (Figs. S5 and S6), reflected by lower Z scores for cytokines (Fig. 4B), chemokines (Fig. 4C), growth factors (Fig. 4D) and all 32 markers (Fig. 4E). *App*^NL-G-F^ mice thus displayed neuroinflammation that was partially attenuated by the hypomorphic PDE4B present in *App*^NL-G-F^/*Pde4b*^Y358C^ mice. Lysates from *App*^NL-G-F^/*Pde4b*^Y358C^ mice also showed significantly decreased levels of IL-9 (Fig. S5K), eotaxin (Fig. S6A), and LIX (Fig. S6C) compared with WT lysates, suggesting additional anti-inflammatory effects of PDE4B inhibition in the brains of 12-month-old mice independent of *App* genotype.

### Modulation of differentially expressed genes in *App*^NL-G-F^ mice by hypomorphic PDE4B

As PDE4B regulates cAMP gradients and ultimately the transcription factor CREB [43], we performed transcriptomic analysis (RNA sequencing) on cerebral cortical tissue from 12-month-old mice to identify molecular pathways potentially underlying the protective effect of PDE4B inhibition in *App*^NL-G-F^ mouse brain. Among 76,076 gene transcripts that were robustly expressed, 531 were differentially expressed (DE) at an adjusted significance threshold of *p* < 0.01 in *App*^NL-G-F^ (527 up, 4 down) (Fig. S7A; Table S3) and 462 were DE in *App*^NL-G-F^/*Pde4b*^Y358C^ (379 up, 83 down) (Fig. S7B; Table S4) versus WT mice. The number of DE transcripts in *App*^NL-G-F^/*Pde4b*^Y358C^ versus *App*^NL-G-F^ mice was 117 (21 up, 96 down) (Fig. S7C; Table S5).

Among the 531 transcripts DE in *App*^NL-G-F^ mice, 314 were also DE in *App*^NL-G-F^/*Pde4b*^Y358C^ versus WT but not significantly different between *App*^NL-G-F^ and *App*^NL-G-F^/*Pde4b*^Y358C^ (Table S6), suggesting transcriptional effects of the *App*^NL-G-F^ allele that were not significantly affected by hypomorphic PDE4B. Included in them are *CCl3* (1 up) encoding MIP-1α (Fig. S8A), corroborating the inflammatory marker assay result, as well as *Itgax* (4 up) (Fig. S8B), a marker of disease-associated microglia (DAM) activation, and *Gfap* (4 up) (Fig. S8C), a marker of astrocyte activation (reactive astrogliosis).

**Fig. 5:**
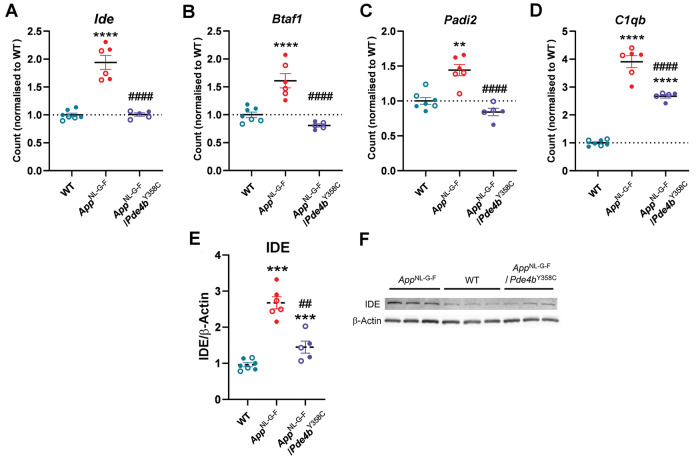
Modulation of differentially expressed cerebral cortical genes in *App*^NL-G-F^ mice by hypomorphic PDE4B. **A** *Ide* (mean of 7 transcripts). **B** *Btaf1* (mean of 3 transcripts). **C** *Padi2* (mean of 2 transcripts). **D** *C1qb* (1 transcript). **E** Immunoreactivity of IDE protein normalized to β-Actin in brains. Genotypic differences were observed (Kruskal-Wallis test, χ^2^_18_ = 14.259, *p* = 0.001). **F** Typical blot of 50 µg protein from brains probed with anti-IDE and anti-β-Actin antibodies. 12-month-old WT (*n* = 7), *App*^NL-G-F^ (*n* = 6) and *App*^NL-G-F^/*Pde4b*^Y358C^ (*n* = 5) mice. Data are plotted as mean ± SEM. **adj. *p* < 0.01, ****adj. *p* < 0.0001 vs. WT; ^####^adj. *p* < 0.0001 vs. *App*^NL-G-F^ for **A**–**D**. ***p < 0.001 vs. WT; ^##^*p* < 0.01 vs. *App*^NL-G-F^ for (**E**).

Among the remaining 217 transcripts DE in *App*^NL-G-F^ mice, all but one (*C1qb*) were not significantly different between *App*^NL-G-F^/*Pde4b*^Y358C^ and WT mice (Table S7). Included in them is *Cxcl5* (1 up) encoding LIX (Fig. S8D), as well as the DAM activation markers *Fth1* (2 up) (Fig. S8E) and *Mamdc2* (4 up) (Fig. S8F), and the reactive astrocyte markers *Dbi* (2 up), *Aqp4* (9 up), *Ifitm3* (1 up) and *Osmr* (6 up) (Fig. S8G–J). Hypomorphic PDE4B had the greatest modulatory effect on 13 transcripts (from four genes), as they were significantly different between *App*^NL-G-F^/*Pde4b*^Y358C^ and *App*^NL-G-F^ mice (blue dots, Fig. S7C; Table S8). These most modulated transcripts include *Ide* (7 up from 7) (Fig. 5A), *Btaf1* (3 up from 5) (Fig. 5B), and *Padi2* (2 up from 2) (Fig. 5C), whose expression was normalized by the hypomorphic PDE4B present in *App*^NL-G-F^/*Pde4b*^Y358C^ mice. Also included is *C1qb* (1 up from 1) (Fig. 5D), a marker of homeostatic microglia, which was significantly different between *App*^NL-G-F^/*Pde4b*^Y358C^ and WT mice, indicating a milder modulatory effect of the PDE4B hypomorph. *App*^NL-G-F^ mice thus displayed gene expression differences that were modulated by the hypomorphic PDE4B present in *App*^NL-G-F^/*Pde4b*^Y358C^ mice.

Among the 75,545 transcripts that were not DE in *App*^NL-G-F^ versus WT mice, 41 were DE in *App*^NL-G-F^/*Pde4b*^Y358C^ (14 up, 27 down) versus both WT and *App*^NL-G-F^ mice (Table S9). *App*^NL-G-F^/*Pde4b*^Y358C^ mice thus displayed gene expression differences driven by hypomorphic PDE4B that were not significantly affected by the *App*^NL-G-F^ allele (e.g., *Casp9* and *Nr1d1*; Fig. S8K, L). A further 106 transcripts were DE in *App*^NL-G-F^/*Pde4b*^Y358C^ (55 up, 51 down) versus WT but not versus *App*^NL-G-F^ mice (Table S9), suggesting modulation by the *App*^NL-G-F^ allele of PDE4B hypomorph-driven gene expression differences (e.g., *Per2*; Fig. S8M).

As increased levels of IDE (insulysin), encoded by *Ide*, have been observed in brain tissue from 9–18-month-old APPswe/PSEN1dE9 [44, 45] and Tg2576 [46] transgenic mice that overexpress human Swedish mutant APP, we quantified the amount of IDE in brain lysates from 12-month-old mice by western blotting. Consistent with the RNA sequencing results, the level of IDE was upregulated in *App*^NL-G-F^ versus WT mice, but this upregulation was blunted in *App*^NL-G-F^/*Pde4b*^Y358C^ lysates (Fig. 5E, F), confirming a modulating effect of hypomorphic PDE4B on IDE expression. Correlation coefficients revealed positive correlations between brain levels of IDE and inflammatory marker Z scores across all genotypes (Fig. S9), indicating that higher levels of IDE were associated with greater inflammation.

## Discussion

Authentic animal models serve as valuable tools for determining the molecular mechanisms of disease progression and testing potential therapeutic approaches. The *App*^NL-G-F^ model develops Aβ plaques, neuroinflammation, damaged synapses, and behavioral and cognitive deficits, while accurately recapitulating endogenous APP expression [29, 30]. However, it was unknown whether *App*^NL-G-F^ mice also replicate the cerebral hypometabolism that is a hallmark feature of AD [39]. Accumulating studies have shown that this decline in cerebral glucose metabolism occurs before pathology and symptoms manifest, continues as symptoms progress, and is more severe than the gradual decline in metabolic efficiency during normal aging [47]. 18-fluorodeoxyglucose positron emission tomography (PET) brain imaging in AD patients has observed cerebral glucose metabolism deficits in a range of brain regions, including the PFC and medial temporal lobe/hippocampus [48, 49], with this hypometabolism being a potentially useful diagnostic biomarker [50].

Using the translational ^14^C-2-DG brain imaging technique, we observed that 13-month-old *App*^NL-G-F^ mice exhibit widespread glucose hypometabolism in multiple brain regions, which was prevented by the hypomorphic PDE4B present in *App*^NL-G-F^/*Pde4b*^Y358C^ mice. Glucose utilization was highest in the thalamus, consistent with the distribution of PDE4B in the primate brain [51, 52]. Targeted inhibition of PDE4B is thus an intervention that increases cerebral metabolism in *App*^NL-G-F^ mice (i.e., improves the neuronal energy state) and therefore has the potential to be disease-modifying in patients. By contrast, acute pretreatment of normal mice and rats with the pan-PDE4 inhibitor rolipram decreases glucose utilization in brain tissue [53–56]. Moreover, pan-PDE4 inhibitors have dose-dependent side effects of nausea and emesis – attributed to the selective inhibition of PDE4D – which limit their tolerability and translational value [57].

The SLM serves as a relay between the entorhinal cortex (EC) and the CA1 and is believed to represent the substrate of distinct aspects of spatial and episodic memory [58, 59]. Neurons of the EC that send projections to the CA1 are the initial degenerating cells in AD [60]. Considering the spatial memory impairment of *App*^NL-G-F^ mice, it is noteworthy that their hippocampal hypometabolism was restricted to the SLM. However, *App*^NL-G-F^ mice also exhibited hypometabolism in the PFC, which itself has a role in spatial memory formation [61, 62]. The restoration of glucose metabolism in both the SLM and PFC thus likely contributed to the improved spatial memory of *App*^NL-G-F^/*Pde4b*^Y358C^ mice.

Cerebral glucose hypometabolism has previously been observed in transgenic APP-overexpressing mouse models of AD. The PDAPP strain showed glucose hypometabolism across multiple brain regions at 10 months of age [63], but this reached statistical significance only in the posterior cingulate cortex in 17-month-old mice following Bonferroni correction for multiple comparisons [64]. The PS/APP strain had localized hypometabolism in several brain regions but an unaltered whole brain average at 16 months [65], whereas the 3xTg-AD strain showed widespread hypometabolism in all measured brain regions at 18 months of age [66].

The area occupied by thioflavin-S-stained Aβ plaques in brain sections from 12-month-old *App*^NL-G-F^ mice was similar to that previously reported for 11-month-old *App*^NL-G-F^ mice, which showed a mild corticolimbic Aβ pathology relative to age-matched 5xFAD and APPswe/PSEN1dE9 mice that overexpress APP [67]. The similar Aβ plaque burdens of *App*^NL-G-F^ and *App*^NL-G-F^/*Pde4b*^Y358C^ mice in the present study suggest that a decrease in Aβ plaque load is not responsible for the prevention of spatial memory and brain metabolism deficits by hypomorphic PDE4B. The situation in *App*^NL-G-F^/*Pde4b*^Y358C^ mice is thus comparable with that of the ∼30% of older adults without signs of cognitive impairment who exhibit the neuropathological features of AD upon autopsy at the time of death [68]. This is not, however, contradictory to PDE4B being a promising therapeutic target for AD. There is a need for non-Aβ-directed therapies for AD because Aβ-targeting antibodies have shown only modest cognitive benefit in early AD [4, 5], and more than 20% of individuals diagnosed with AD do not have Aβ plaque burden as assessed by PET imaging [69].

Despite the central role of PDE4B in inflammation [70–72], the upregulation of multiple inflammatory markers in both *App*^NL-G-F^/*Pde4b*^Y358C^ and *App*^NL-G-F^ mice suggests that the protective effect of hypomorphic PDE4B cannot be attributed to a broad anti-inflammatory response. Although TNFα levels were not significantly affected by genotype, the lack of detectable TNFα in four of the five *App*^NL-G-F^/*Pde4b*^Y358C^ mice is consistent with the known suppression of TNFα production by PDE4B ablation and selective inhibition [72, 73].

RNA sequencing to identify molecular pathways potentially underlying the protective effect of hypomorphic PDE4B revealed that 531 of 76,076 robustly expressed gene transcripts were DE in cerebral cortical tissue from *App*^NL-G-F^ versus WT mice. None of the PDE4 transcripts (*Pde4a*–*d*) were DE in *App*^NL-G-F^ mice (Table S10), unlike the upregulation of *Pde4b* in cultured microglia exposed to Aβ peptides [11]. Among APP-overexpressing mouse models, the 3xTg-AD strain exhibited increased PDE4B and PDE4D protein levels in hippocampus and PFC, which were decreased by rolipram [22], and the APPswe/PSEN1dE9 strain exhibited increased PDE4B and PDE4D protein levels in cerebral cortex, which were decreased by roflumilast [24].

Among the transcripts DE in *App*^NL-G-F^ mice, 59% were also DE in *App*^NL-G-F^/*Pde4b*^Y358C^ versus WT mice, including the upregulation of *CCl3*, encoding MIP-1α that was also upregulated in the inflammatory marker assay. None of the *Pde4b* transcripts (0 of 9) were DE in *App*^NL-G-F^/*Pde4b*^Y358C^ versus WT mice (Table S10), as expected from prior analyses of *Pde4b*^Y358C^ mouse brain tissue [28]. Both increased and decreased expression of *Pde4b* in the hippocampus have been shown to impair contextual fear memory in mice [74].

Remarkably, the levels of only 13 of the DE transcripts – from four genes: *Ide*, *Btaf1*, *Padi2,* and *C1qb* – were significantly different between *App*^NL-G-F^/*Pde4b*^Y358C^ and *App*^NL-G-F^ mice, suggesting that their expression was the most modulated by the hypomorphic PDE4B. The 13 include all *Ide* (7 of 7), *Padi2* (2 of 2), and *C1qb* (1 of 1) transcripts, but only three of the five *Btaf1* transcripts in the RNA sequence dataset. Multiple independent transcripts thus provide support for three of these genes at the conservative adjusted significance threshold employed (*p* < 0.01), but we cannot exclude the possibility that the single transcript representing *C1qb* is a false positive.

*Padi2*, encoding protein-arginine deiminase type-2 (PADI2) that converts arginine residues in proteins into citrullines (citrullination) through deamination [75], and *Ide*, encoding a ubiquitously expressed metalloprotease (IDE) that cleaves peptides including Aβ [76, 77], are noteworthy because their expression is upregulated in postmortem AD patient brains compared with age-matched controls. Markedly increased levels of PADI2 are detected in hippocampal samples from AD patients, and the immunoreactivity of PADI2 and citrullinated proteins coincides with glial fibrillary acidic protein (GFAP)-positive astrocytes [78]. Increased levels of IDE are detected in postmortem brains from patients with moderate AD pathology (Braak stages III–IV) although decreased levels of IDE are found in severe AD (Braak stages V–VI) [79].

IDE levels in cerebral cortex from 9-month-old APPswe/PSEN1dE9 mice are elevated after the formation of the first Aβ plaques and show a positive correlation with full-length APP levels [44]. In 10- and 18-month-old APPswe/PSEN1dE9 mice, the expression of IDE is inversely correlated with spatial memory in the Morris water maze [45]. In 16-month-old Tg2576 mice, increased IDE expression appears within GFAP-positive astrocytes surrounding Aβ plaques [46]. Like these transgenic APP-overexpressing strains, 12-month-old *App*^NL-G-F^ mice, but not *App*^NL-G-F^/*Pde4b*^Y358C^ mice, show upregulation of IDE protein in the brain – conceivably representing a compensatory mechanism aimed to decrease Aβ, MIP-1α, and MIP-1β levels. Despite the involvement of GFAP-positive astrocytes in the expression of *Padi2* and *Ide*, this cell type is unlikely to mediate the modulation of these transcripts by PDE4B inhibition because *Gfap* was upregulated in both *App*^NL-G-F^ and *App*^NL-G-F^/*Pde4b*^Y358C^ mice. However, the upregulation of some less abundant reactive astrocyte markers, such as *Aqp4*, was blunted in *App*^NL-G-F^/*Pde4b*^Y358C^ mice.

The normalization of IDE expression in *App*^NL-G-F^/*Pde4b*^Y358C^ mice suggests that activation of the cAMP signaling pathway negatively regulates IDE expression. This observation is consistent with the finding that IDE expression in streptozotocin-treated APPswe/PSEN1dE9 mice is decreased by administration of the cAMP analog and non-subtype-selective cAMP phosphodiesterase inhibitor bucladesine in a dose-dependent manner [80]. The convergence between our observations in *App*^NL-G-F^ mice and published findings from transgenic mice that overexpress APP suggest that cerebral hypometabolism and IDE upregulation are not artifacts related to the non-physiological overproduction of various APP fragments in the APP-overexpressing strains [30].

Although we have identified that the *Pde4b*^Y358C^ hypomorph corrected expression changes of specific cortical transcripts but did not decrease Aβ plaque burden in *App*^NL-G-F^ mice, a limitation of this study is that it has not elucidated the mechanism underlying the protective effect of hypomorphic PDE4B in the *App*^NL-G-F^ model. Since we aimed to evaluate the neurocognitive effects of genetically inhibiting PDE4B in *App*^NL-G-F^ mice, the study did not include a group with the *Pde4b*^Y358C^ mutation on a WT (*App*^+/+^) background. Previous studies have employed similar experimental designs without a treatment-only *App*^+/+^ group to assess the effects of genetic modifications in *App*^NL-G-F^ mice [81–83]. However, the lack of a *Pde4b*^Y358C^-only group precluded statistical analysis of *Pde4b* genotype as an independent variable. Consequently, an additional limitation is that we could not evaluate the effect of PDE4B inhibition in 12-month-old mice independently of the *App*^NL-G-F^ mutant allele. Another limitation is that the *App*^NL-G-F^ model does not exhibit tau-containing neurofibrillary tangles, a histopathological hallmark of AD [29, 30]. Rolipram suppresses tau phosphorylation in 11-month-old 3xTg-AD mice [22] and in the rTg4510 mouse model of frontotemporal dementia at 3–4 months of age [84], but no work has been published on the effects of PDE4B subtype-specific inhibition on tau pathology. A further limitation is that the B6.C-*Pde4b*^enu1H^ mouse line employed does not permit spatial or temporal control over expression of the *Pde4b*^Y358C^ mutation. A *Pde4b*^Y358C^ conditional knock-in line would allow us to test whether hypomorphic PDE4B can arrest or reverse progression at different stages of disease in the *App*^NL-G-F^ model.

In summation, our data show that *App*^NL-G-F^ mice exhibit spatial memory and brain metabolism deficits that are prevented by targeted inhibition of PDE4B, a cAMP hydrolyzing enzyme that has been implicated in the pathogenesis of AD by a recent meta-analysis of GWAS data [7]. To the best of our knowledge, this is the first study demonstrating that PDE4B subtype-specific inhibition has a protective effect in a preclinical model of AD. This novel finding identifies the PDE4B subtype as a key regulator of disease manifestation in the *App*^NL-G-F^ model and a promising therapeutic target for AD.

### Supplementary information

Supplementary Information

Table S1

Table S3

Table S4

Table S5

Table S6

Table S7

Table S9

Table S10
