# Supplementary material for: Protective effect of PDE4B subtype-specific inhibition in an App knock-in mouse model for Alzheimer’s disease
Source: Neuropsychopharmacology. 2024 Mar 23;49(10):1559–68. doi: 10.1038/s41386-024-01852-z (PMC11319650; doi:10.1038/s41386-024-01852-z)
Supplement: Supplementary file 1 — Supplementary Information [file 41386_2024_1852_MOESM1_ESM.doc]

Supplementary Information for

**Protective Effect of PDE4B Subtype-specific Inhibition in an *App* Knock-in Mouse Model for Alzheimer’s Disease**

Paul Armstrong^†^, Hüseyin Güngör^†^, Pariya Anongjanya, Clare Tweedy, Edward Parkin, Jamie Johnston, Ian M. Carr, Neil Dawson and Steven J. Clapcote*

^†^These authors contributed equally to this work.

*Correspondence: s.j.clapcote@leeds.ac.uk

**SUPPLEMENTARY METHODS AND MATERIALS**

**Mice**

The C57BL/6-*App*^tm3.1(NL-G-F)Tcs^ (*App*^NL-G-F^) line [1] from the RIKEN BioResource Research Center and the B6.C-*Pde4b*^enu1H^ (*Pde4b*^Y358C^) line [2] were maintained by backcrossing to C57BL/6JCrl (Charles River) for at least 10 generations. *App*^NL-G-F^ heterozygotes (*App*^NL-G-F/+^) were crossed with *Pde4b*^Y358C^ heterozygotes (*Pde4b*^Y358C/+^) to yield *App*^NL-G-F/+^;*Pde4b*^Y358C/+^ double heterozygotes that were intercrossed to generate *App*^+/+^;*Pde4b*^+/+^ (wild-type; WT), *App*^NL-G-F/NL-G-F^;*Pde4b*^+/+^ (*App*^NL-G-F^) and *App*^NL-G-F/NL-G-F^;*Pde4b*^Y358C/Y358C^ (*App*^NL-G-F^/*Pde4b*^Y358C^) littermates for phenotypic testing. Pups were weaned at 4 weeks of age and grouped housed (3–5 mice/cage) with same-sex littermates under a 12-h light/dark cycle (lights on at 06:00). Pelleted feed (CRM-P, SDS Diets) and water were provided *ad libitum*. The mouse experiments were conducted in accordance with the UK Animals (Scientific Procedures) Act 1986 under UK Home Office licences and approved by institutional Animal Welfare and Ethical Review Bodies at the University of Leeds and Lancaster University.

**Genotyping**

DNA was extracted using proteinase K (Sigma-Aldrich) from ear biopsies taken at weaning. Mice were genotyped for the *App*^NL-G-F^ allele (specifically the I716F ‘Iberian’ mutation) on chromosome 16 by the absence of a *Bsa*BI restriction site in a 470-bp DNA fragment amplified using primers forward: 5'-CTG TTA AAG GGC TTC AGA TCC-3' and reverse: 5'-AGG GAT GTT GCT TTT CTC CTA-3'. Mice were genotyped for the *Pde4b*^Y358C^ allele on chromosome 4 by the presence of a *Bsi*HKAI restriction site in a 443-bp fragment amplified using primers forward: 5'-ACC TGC CTT TGA AAG TAG CAT-3' and reverse: 5'-AGC TCT GTT CCA AGA TAA TCG-3' [2]. The thermocycling program was 95°C for 3 min, followed by 35 cycles of 94°C for 30 s, 58°C (*App*^NL-G-F^) or 59°C (*Pde4b*^Y358C^) for 60 s, 72°C for 40 s, followed by 72°C for 10 min. PCR products were visualized using agarose gel electrophoresis with ethidium bromide staining.

**Barnes maze**

The Barnes maze test was conducted on mice at 12 months of age according to a protocol adapted from a previously described method [3]. Mice were handled for one week prior to behavioral testing and were transferred to the experimental room 30 min prior to the start of testing. The apparatus was cleaned with 70% ethanol between each mouse. Experiments were conducted between 9 am and 5 pm. A webcam was positioned directly above the apparatus to record the trials using ANY-maze Video Tracking Software (Stoelting). Experimenters were blinded to genotype during behavioral testing.

The maze consisted of a 9 mm thick satin white PVC circular arena (122 cm diameter) with 20 equidistant holes (5 cm diameter) around the perimeter (7.5 cm from the edge). The maze had no perimeter walls and was raised 40 cm from the ground. The maze was surrounded by various distal spatial cues. Overhead room lights and two 60 w lamps illuminated the maze to provide an aversive stimulus to escape. An escape box (25 x 6 x 5 cm) was attached to the underside of the maze, allowing escape from the arena via one of the 20 holes (the target hole).

Mice initially received a 2-min habituation session to the maze and escape box. The training phase consisted of 20 trials over 5 days (4 trials/day). The maximum duration of each trial was 120 s with a 1-h inter-trial interval. For each mouse, the escape box location (north, south, east or west hole, balanced between genotypes) remained constant throughout training. Immediately preceding each trial, the mouse was placed onto the center of the arena enclosed in a cardboard chamber. Once the chamber was removed, the mouse had free access to the entire arena for 120 s. If the mouse located and entered the escape box within the 120 s, it was allowed to spend 60 s inside before being returned to the home cage. If the mouse failed to enter the escape box within 120 s, the experimenter manually guided it to the target hole.

During training trials, the following parameters were used to assess spatial learning: latency (s) of the mouse’s head to enter the target hole for the first time (primary latency); distance travelled (m) before the mouse made its first head entry into the target hole (primary path length); and number of head entries into incorrect holes before the first target hole entry (primary errors). For primary latency, the maximum time of 120 s was recorded if a mouse did not find the target hole. For primary path length, the total distance travelled in the trial was recorded if a mouse did not find the target hole, although the data point was excluded from the analysis if a mouse travelled less than 0.3 m. For primary errors, the total number of head entries into incorrect holes was recorded if a mouse failed to find the target hole by the end of the trial. The speed travelled during the training trials (velocity; m/s) was also measured. Measurements were averaged across the four trials per day.

Seventy-two hours after the last training day, a probe trial was conducted, during which the escape box was removed and the mouse was allowed to explore the arena for 120 s. Three measures were used to assess spatial memory during the probe trial: time spent in the target quadrant (a geometric area covering 25% of the arena with the escape hole in the center of five holes); time spent in the target sector (a geometric area covering 5% of the arena for each of the 20 holes); and number of head entries to the target hole annulus (a 7 cm diameter circle centered on the target hole).

Statistical analysis was performed using SPSS and GraphPad Prism. Data were tested for normality using the Shapiro-Wilk test, and for homoscedasticity using Levene’s test. We used Student’s *t* tests (with Welch corrections for heteroscedasticity), and two-way repeated-measures analysis of variance (ANOVA) when data passed normality and homoscedasticity assumptions. If applicable, the Greenhouse–Geisser adjustment was used to correct for violations of sphericity. Significant ANOVA interactions were analysed further using simple main effect analysis with Fisher’s least significant difference. If the data violated normality, it was square root transformed or Kruskal-Wallis tests and Friedman’s ANOVA were used. Unless otherwise stated, α was set at 0.05. Graphs were prepared using GraphPad Prism version 6.

**^14^C-2-DG autoradiography**

Brain specimens were prepared from the same mice as used earlier for the Barnes maze test. The ^14^C-2-deoxyglucose (^14^C-2-DG) functional brain imaging technique [4, 5] was used to map cerebral metabolism, in terms of tissue glucose utilization, across 39 regions of interest (RoI) in mice aged 13 months. Mice were injected intraperitoneally (i.p.) with 4.625 MBq/kg of ^14^C-2-DG (American Radiolabelled Chemicals Inc.) in sterile physiological saline at a steady rate over a 10-s period. Animals were then returned to a home cage. At exactly 45 min after isotope injection, animals were decapitated, and a terminal blood sample collected by torso inversion into heparinized weigh boats. Plasma glucose levels (mmol/L) were measured directly from un-heparinized torso blood using a glucometer (Accu-chek Aviva). The brain was rapidly dissected out intact, then frozen in isopentane (-40°C) and stored at -80°C until sectioning. Blood samples were centrifuged to separate the plasma and 10 µl aliquots removed for the determination of plasma ^14^C levels, in triplicate, by liquid scintillation analysis (Packard). Frozen brains were then sectioned (20 µm) in the coronal plane in a cryostat (-20°C). A series of three consecutive sections were retained from every 120 µm, thaw mounted onto glass cover slips and rapidly dried on a hot plate (70°C).

Autoradiograms were generated by apposing these sections together with pre-calibrated ^14^C-standards (39–1069 nCi/g tissue equivalents, American Radiolabelled Chemicals Inc.) to X-ray film (Carestream Biomax MR Film, Sigma-Aldrich) for 7 days. Autoradiographic images were analyzed by a computer-based image analysis system (MCID/M5+). The local isotope concentration for each brain RoI was derived from the optical density of autoradiographic images relative to that of the co-exposed ^14^C-standards. Measurements were taken from 39 anatomically distinct RoI, including subfields of the prefrontal cortex (PFC), septum, basal ganglia, hippocampus and other cerebral cortical regions, defined with reference to a stereotactic mouse atlas [6]. The rate of local tissue glucose utilization (pmol/g/min) was calculated based on the tissue ^14^C concentration with reference to the specific activity of the ^14^C-2-DG (55 mCi/mmol) and the 45-min experimental time window. Data were analyzed by unpaired (independent) Student's *t* test with Bonferonni-Holm post hoc correction for multiple comparisons, with α set at 0.05.

The approximate bregma measurements for the three sections shown in Supplementary Fig. S2 are estimates based on the anatomy of the section and reference to the Allen Reference Atlas – Mouse Brain [7] rather than direct measurements.

**Thioflavin-S staining**

At 12 months of age, WT (*n* = 4 [2♂, 2♀]), *App*^NL-G-F^ (*n* = 6 [3♂, 3♀]) and *App*^NL-G-F^/*Pde4b*^Y358C^ (*n* = 6 [3♂, 3♀]) mice were terminally anesthetized with 20% sodium pentobarbital (Pentoject; Animalcare) and intracardially perfused with 30 ml of 0.1 M phosphate buffered saline followed by 30 ml of 4% paraformaldehyde (PFA). After perfusion, the brain was incubated in 4% PFA overnight at 4°C, embedded in paraffin blocks, and sectioned at approximately -1.58 mm from bregma (showing cerebral cortex and hippocampus) to a thickness of 4 μm using a microtome (Leica). To stain Aβ deposits, sections were incubated in filtered 1% aqueous thioflavin-S (Toronto Research Chemicals) for 7 min, and washed three times with 80% ethanol. Stained sections were dehydrated, cleared, and mounted onto slides in fluorescence-free mounting medium. Fluorescence images were obtained with an AxioScan Z1 fluorescent slide scanner (Carl Zeiss). To measure plaque load, the percentage of surface area occupied by thioflavin-S-stained Aβ plaques on *n* = 2 sections/mouse was analyzed using Fiji software.

**Inflammation assay**

At 12 months of age, WT (*n* = 7 [4♂, 3♀]), *App*^NL-G-F^ (*n* = 6 [3♂, 3♀]) and *App*^NL-G-F^/*Pde4b*^Y358C^ (*n* = 5 [2♂, 3♀]) mice were euthanized via cervical dislocation and their brains was quickly extracted, hemisected and snap frozen in liquid N_2_. The right hemisphere was stored at -80°C for RNA sequencing. The left hemisphere was homogenized with a rotor-stator homogenizer (TissueRuptor; Qiagen) in RIPA lysis buffer containing containing PMSF, sodium orthovanadate, protease inhibitors (sc-24948; Santa Cruz), and phosphatase inhibitors (PhosSTOP; Roche). The homogenate was cleared by centrifugation at 10,000× *g* for 10 min at 4°C and the total protein concentration of the supernatant was determined by BCA protein assay (Pierce). Protein samples (2.5 mg in 100 µl) were analyzed for the following inflammatory markers in duplicate using the Mouse Cytokine/Chemokine 32-Plex Array (Eve Technologies): cytokines (G-CSF, GM-CSF, IFNγ, IL-1α, IL-1β, IL-2, IL-3, IL-4, IL-5, IL-6, IL-9, IL-10, IL-12p40, IL-12p70, IL-13, IL-15, IL-17A, LIF, M-CSF, TNFα), chemokines (Eotaxin, IP-10, LIX, MCP-1, MIG, MIP-1α, MIP-1β, MIP-2, RANTES) and growth factors (IL-7, KC, VEGF-A). The remaining supernatant was stored at -80°C for western blotting.

The level of each inflammatory marker (pg/ml) was averaged across the duplicates of each sample and then analysed using either a two-way ANOVA, with genotype and sex as between subject factors, if the data were normally distributed, or a Kruskal-Wallis test to compare genotypes and a Mann-Whitney *U* test to compare sexes if the data were non-parametric. A Z-score was calculated for each inflammatory marker and averages were calculated to obtain a total composite Z-score (all 32 markers), a cytokine composite Z-score (20 cytokines), a chemokine composite Z-score (9 chemokines) and a growth factor Z-score (3 growth factors).

**RNA sequencing**

RNA was extracted from cerebral cortical samples (frontal pole to -1.5 mm from bregma) dissected from the right hemispheres previously removed from the WT (*n* = 7), *App*^NL-G-F^ (*n* = 6) and *App*^NL-G-F^/*Pde4b*^Y358C^ (*n* = 5) mice killed for the inflammation assay and stored at -80°C. The concentration and integrity of the RNA sample were assessed using an Invitrogen Qubit RNA assay (Agilent) and a BioAnalyzer 2100 (Agilent), respectively. For the library preparation, 500 ng of total RNA were used. The libraries were prepared using an IDT xGen Broad-Range Library Prep Kit (IDT) according to the manufacturer’s protocol. During this process, the libraries were indexed using xGen UDI 10nt Primers (97-192 – Set 2) (IDT). The prepared libraries were quantified via a fluorometric method involving a QuantiFluor dsDNA assay (Promega) and qualified using electrophoretic separation on a TapeStation 4200 (Agilent). The libraries were normalized and pooled before sequencing on a NovaSeq 6000 (Illumina) instrument

Sequence data in fastq format were quality checked using FastQC software [8] before and after reads were trimmed of poor quality bases (Phred quality score < 20) and contaminating adapter sequences by Cutadapt software [9], with trimmed reads less than 30 nucleotides discarded. Reads were aligned to mouse genome (GRCm39/mm39) reference sequences from the UCSC Genome Browser database [10] using the splicing-aware STAR aligner [11]. Reads were aligned with reference to known splice junctions of the RefSeq gene annotation dataset, obtained from the UCSC database using the Table Browser tool [12]. The resulting alignments were checked for quality using QualiMap software [13]. Picard tools [14] was then used to mark PCR/Optical duplicate read alignments and the resultant BAM files were indexed using Samtools software [15] and visualized using IGV browser [16] to check for genomic DNA contamination and the presence of PCR duplicates.

The Bioconductor R package Rsubread [17] was used to determine the number of reads aligned to each transcript in the RefSeq mm39 annotation dataset. Multi-mapping read pairs were counted as a fraction of all equivalent alignments. Since the level of PCR duplicates was low, read count data was generated with their inclusion. This data was imported into the R package DeSeq2 [18] to identify differentially expressed transcripts, with those with a *p*-value Benjamini-Hochberg adjusted for multiple testing [19] of < 0.01 retained and linked to the appropriate gene name using the ‘bitr’ function of the clusterProfiler R package [20].

**Western blotting**

The same brain lysates that were prepared for the inflammation assay were used for western blotting. Samples of 50 µg total protein were prepared for gel loading by the addition of Laemmli sample buffer (Bio-Rad) with 5% β-mercaptoethanol, and incubated at 95°C for 5 min. Samples were separated by SDS-polyacrlyamide gel electrophoresis on 4–15% Mini-PROTEAN TGX gels (Bio-Rad) in parallel with 5 μl Amersham ECL Rainbow Marker (Cytiva), and then blotted (1.5 h, 100 V) onto Amersham Hybond PVDF membrane (Cytiva). The membrane was then blocked with 5% skimmed milk (Marvel) in PBST (PBS, 0.05% Tween-20) for 1 h at room temperature (RT).

After blocking, the membrane was incubated with rabbit polyclonal antibody PC730 to insulin-degrading enzyme (IDE) (1:2,000, Millipore) in 5% milk in PBST overnight at 4°C. Following incubation, membranes were washed with PBST three times for 10 min. Membranes were then incubated with horseradish peroxidase (HRP)-conjugated swine polyclonal anti-rabbit secondary IgG (1:2,000, Dako) in 5% milk in PBST for 2 h at RT. Following incubation, membranes were washed three times with PBST. Immune complexes were visualized by enhanced chemiluminescence (Amersham ECL Prime, Cytiva) using an iBright CL1500 imaging system (Invitrogen).

To confirm equal loading, the membrane was immersed in Restore Western Blot Stripping Buffer (Pierce) for 15 min at RT before being washed twice with PBST. The membrane was then blocked with 5% milk in PBST overnight at 4°C, washed three times with PBST, and incubated with mouse monoclonal antibody AC-15 to β-actin (1:4,000, Sigma-Aldrich) for 2 h at RT. Following incubation, the membrane was washed three times with PBST and incubated with HRP-conjugated rabbit polyclonal anti-mouse secondary IgG (1:1,000, Dako) for 2 h at RT. Each western blot was performed in triplicate. Densitometry analysis to quantify the visualized bands was carried out using ImageJ 1.51 [21]. IDE levels were normalized to the β-actin loading control for each sample. The normalized IDE/β-actin data were converted to Z-scores and then Spearman's rank correlation coefficients were calculated between them and the four Z-score inflammatory composite scores (see above).

**SUPPLEMENTARY TABLES**

**Table S2. Plasma ^14^C-2-deoxyglucose and glucose levels in WT, *App*^NL-G-F^ and *App*^NL-G-F^/*Pde4b*^Y358C^ mice**

| Parameter | WT | *App*^NL-G-F^ | *App*^NL-G-F^/*Pde4b*^Y358C^ |
| --- | --- | --- | --- |
|  |  |  |  |
| Plasma ^14^C-2-deoxyglucose (µCi/ml) | 34.87 ± 1.82 | 29.17 ± 2.46 | 32.55 ± 2.46 |
| Plasma Glucose (mmol/L) | 7.99 ± 0.31 | 8.73 ± 0.38 | 9.03 ± 0.34 |

Data shown as mean ± SEM. Plasma parameters were not significantly different between the different genotypes (*t* test with Bonferroni correction for multiple comparisons).

**Table S8. Differentially expressed cerebral cortical transcripts in *App*^NL-G-F^ most modulated by PDE4B inhibition in *App*^NL-G-F^/*Pde4b*^Y358C^**

| RefSeq | Gene symbol | Encoded protein | Protein function ^[22]^ | *App*^NL-G-F^ vs. WT | *App*^NL-G-F^/*Pde4b*^Y358C^ vs. WT | *App*^NL-G-F^/*Pde4b*^Y358C^ vs. *App*^NL-G-F^ |
| --- | --- | --- | --- | --- | --- | --- |
| XM_036161446, XM_036161445, XM_036161444, XM_036161443, XM_036161442, XM_036161441, NM_031156 | *Ide* | Insulin-degrading enzyme (insulysin) | Degradation of peptides including insulin, glucagon, Aβ and MIP-1α/β. | > | = | < |
| NM_001080706, XM_006526561,  XM_036161373  [XM_017318039, XM_030250684]^†^ | *Btaf1* | B-TFIID TATA-box binding protein associated factor 1 | Regulation of transcription in association with TATA binding protein (TBP). | > | = | < |
| NM_008812, XM_006538632 | *Padi2* | Peptidylarginine deiminase 2 | Catalysis of the deimination of arginine residues of proteins. | > | = | < |
| NM_009777 | *C1qb* | Complement C1q B chain | B-chain polypeptide of C1q involved in the classical complement cascade. | > | > | < |

>, significantly increased; <, significantly decreased; = no significant difference

^†^, two *Btaf1* transcripts [in square brackets] were not significantly different in *App*^NL-G-F^ v WT mice

**SUPPLEMENTARY FIGURES**


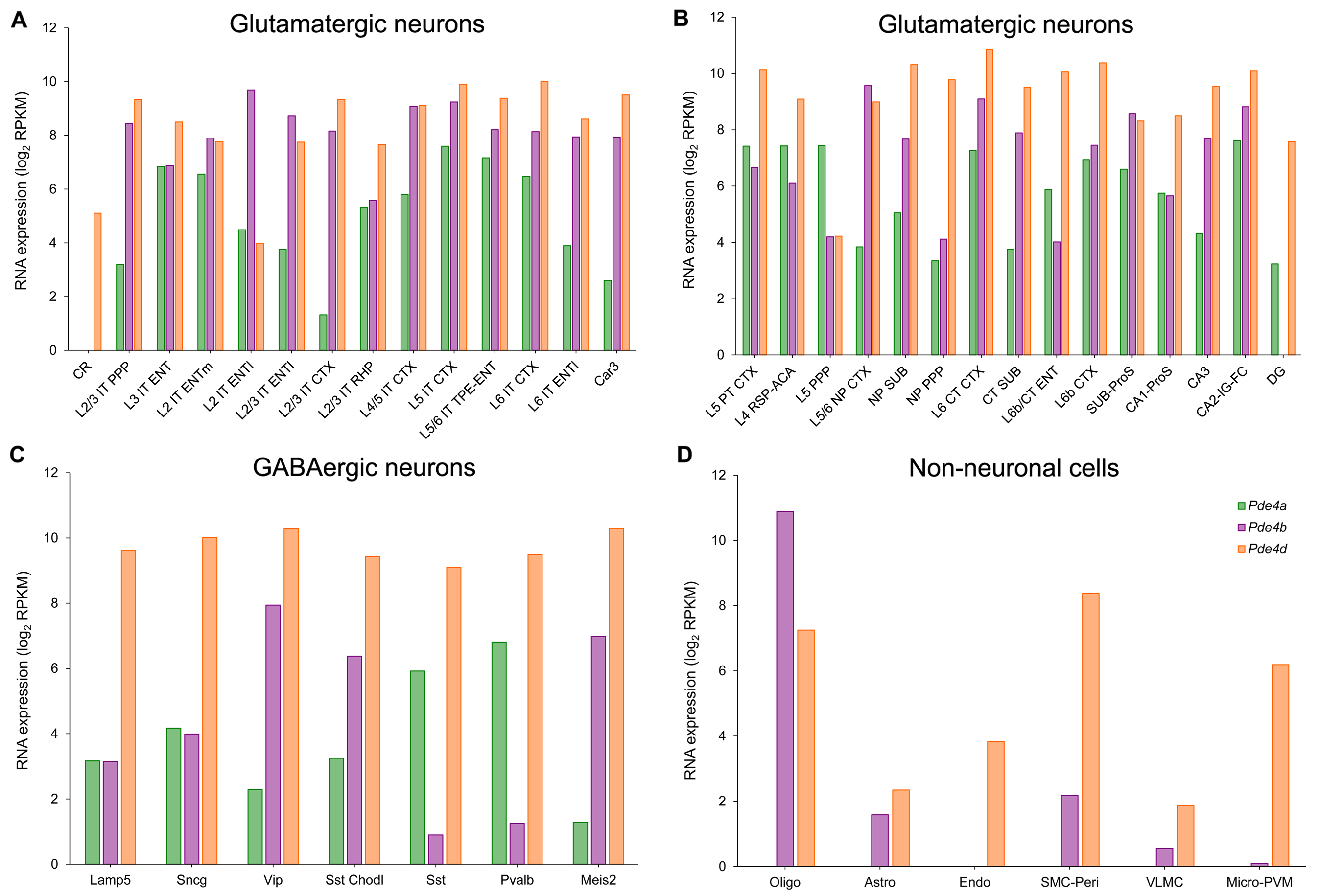


**Fig. S1. *Pde4a*, *Pde4b* and *Pde4d* mRNA expression in different cell types in adult mouse cerebral cortex and hippocampus.** Single-cell RNA sequencing data from the Allen Cell Types Database for *Pde4a*, *Pde4b*, and *Pde4d* among 42 types of glutamatergic and GABAergic neurons and non-neuronal cells in cerebral cortex and hippocampal formation from adult (postnatal day P53-P59) mice, presented as trimmed mean log_2_ CPM (counts per million reads mapped), averaged after excluding the 25% highest and 25% lowest expression values. *Pde4c* transcripts were not identified in any cell type [23, 24].


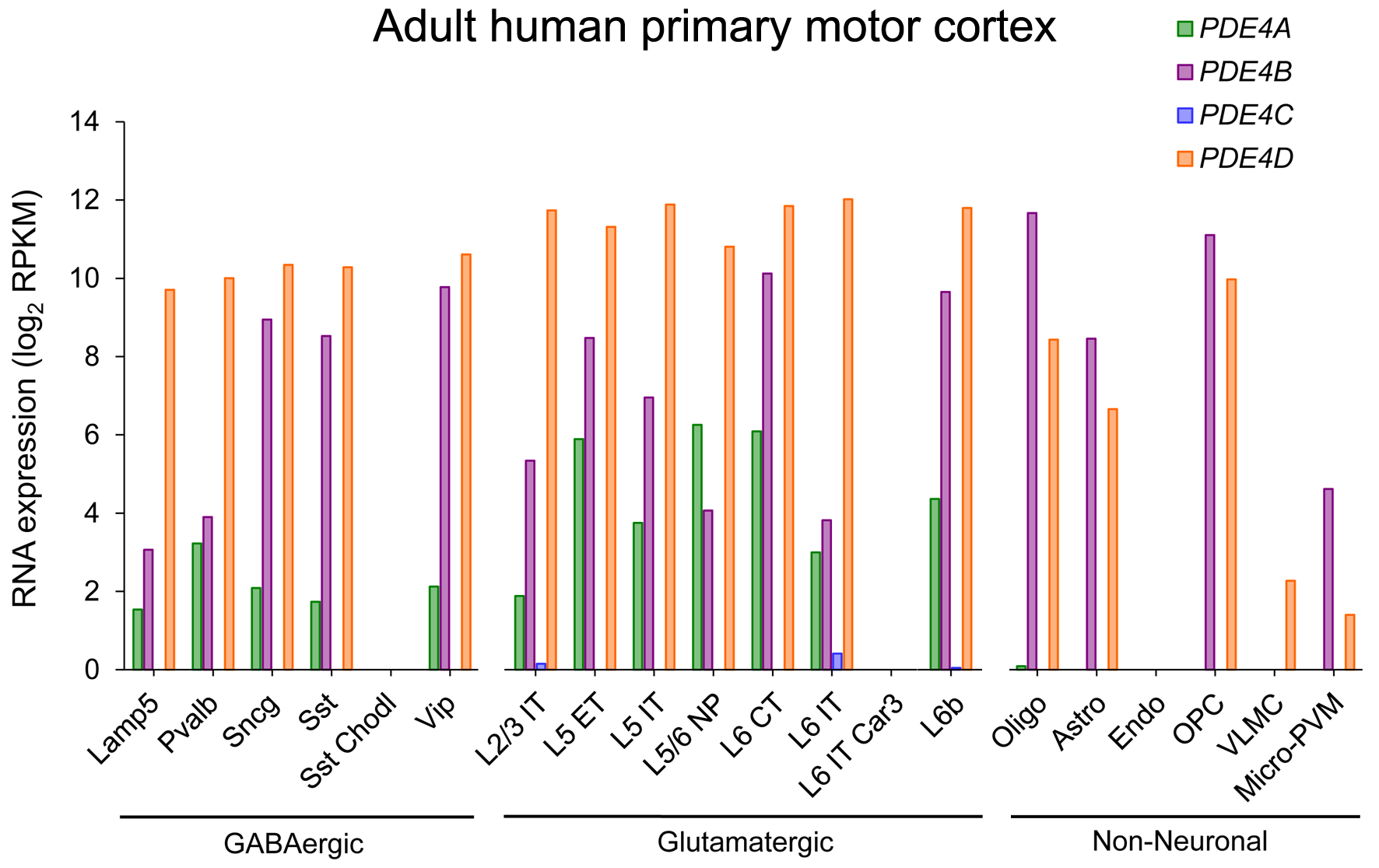


**Fig. S2. *PDE4A*, *PDE4B*, *PDE4C* and *PDE4D* mRNA expression in different cell types in adult human primary motor cortex.** RNA-sequencing data from the Allen Cell Types Database for *PDE4A*, *PDE4B*, *PDE4C* and *PDE4D* among 20 types of GABAergic and glutamatergic neurons and non-neuronal cells, presented as trimmed mean log_2_ CPM, averaged after excluding the 25% highest and 25% lowest expression values [25, 26].


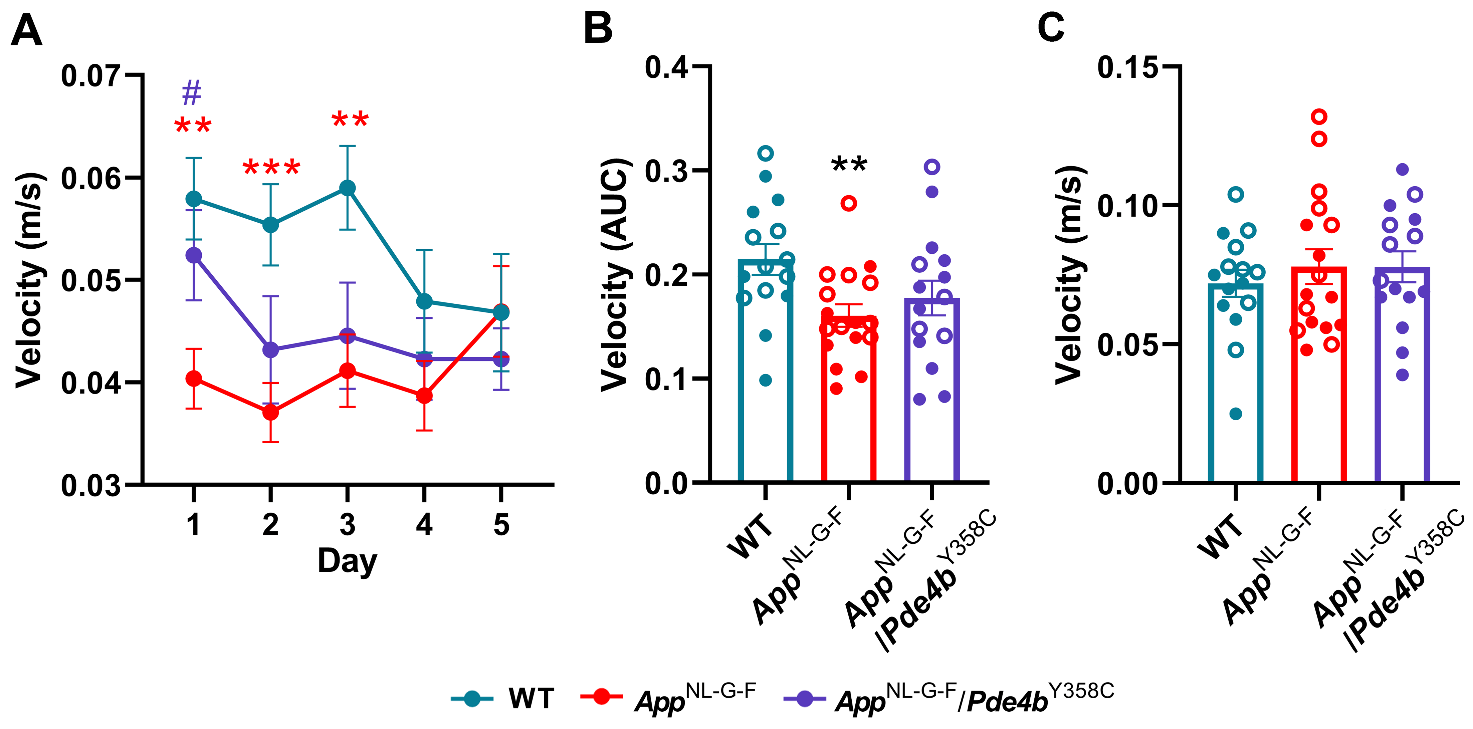


**Fig. S3. Velocity of WT, *App*^NL-G-F^ and *App*^NL-G-F^/PDE4B^Y358C^ mice during training and probe trials in the Barnes maze.** **A** Velocity (m/s) during training trials. ANOVA revealed main effects of day (*F* = 3.677, *p* = 0.024), genotype (*F*_2,41_ = 3.610, *p* = 0.036), and day*genotype interaction (*F* = 2.93, *p* = 0.02). **B** Velocity area under the curve (AUC) during training trials. ANOVA revealed a main effect of genotype (*F*_2,41_ = 3.952, *p* = 0.027). **C** Velocity (m/s) during the probe trial. ***p* < 0.01; ****p* < 0.001 vs. WT. ^#^*p* < 0.05 vs. *App*^NL-G-F^. Open circles, females; closed circles, males.


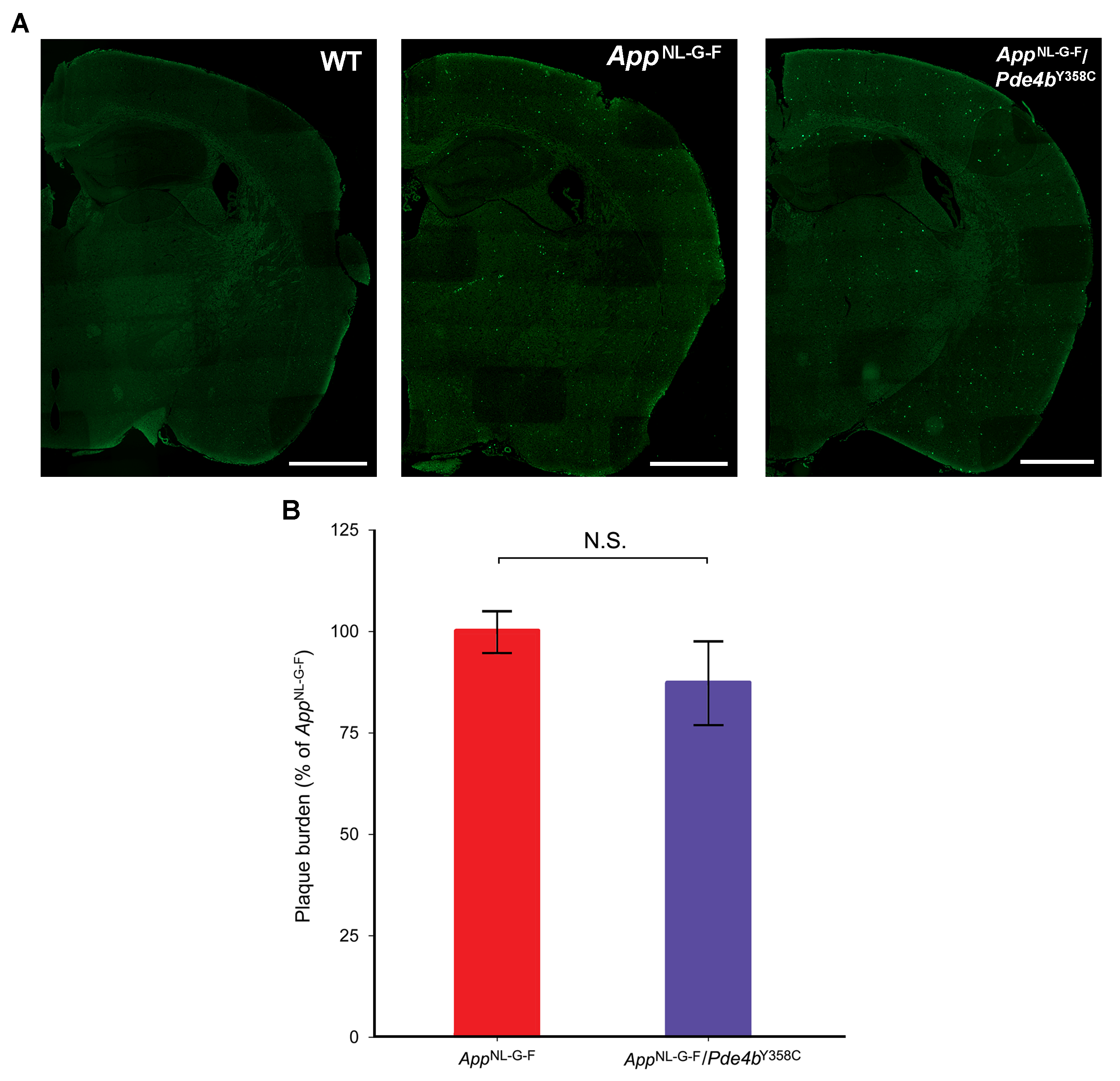


**Fig. S4. Aβ deposition stained by thioflavin-S in brain sections from 12-month-old WT, *App*^NL-G-F^ and *App*^NL-G-F^/*Pde4b*^Y358C^ mice.** **A** Exemplar images of thioflavin-S staining in brain sections at approximately -1.58 mm from bregma, showing cerebral cortex and hippocampus. Scale bars: 1 mm. **B** Quantitative analysis of the occupied area of Aβ plaques stained by thioflavin-S in brain sections from *App*^NL-G-F^ (*n* = 6) and *App*^NL-G-F^/*Pde4b*^Y358C^ (*n* = 6) mice (one-way ANOVA: genotype, *F*_1,10_ = 1.21, *p* = 0.298). The final value for each mouse was obtained by calculating the mean of *n* = 2 thioflavin-S stained sections/mouse (*n* = 6 mice/genotype; *n* = 12 sections/genotype). N.S., not significantly different.


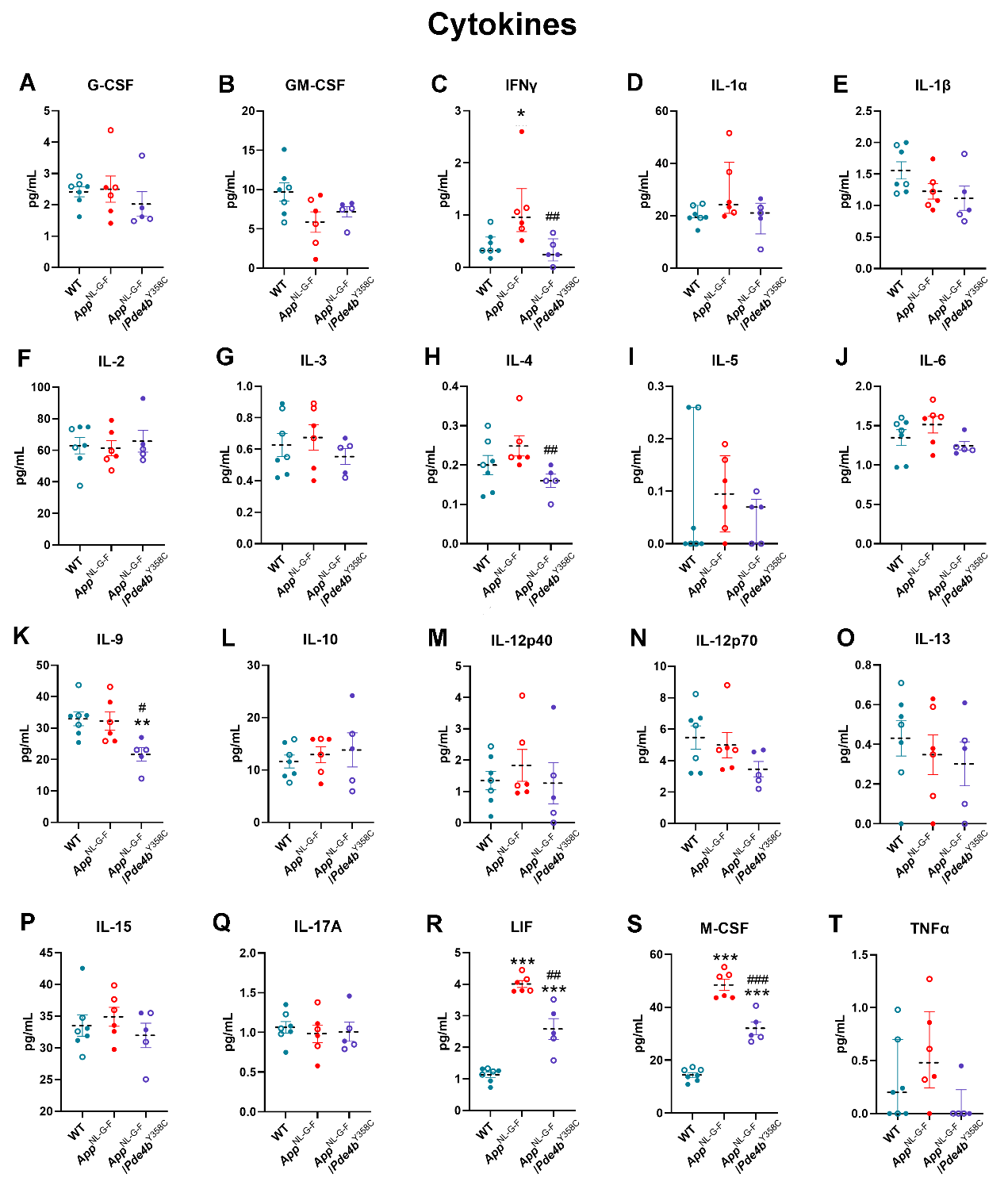


**Fig. S5. Cytokine marker levels in brains from 12-month-old WT (*n* = 7), *App*^NL-G-F^ (*n* = 6) and *App*^NL-G-F^/*Pde4b*^Y358C^ (*n* = 5) mice.** **p* < 0.05; ***p* < 0.01; ****p* < 0.001 vs. WT. ^##^*p* < 0.01; ^###^*p* < 0.001 vs. *App*^NL-G-F^. Open circles, females; closed circles, males.


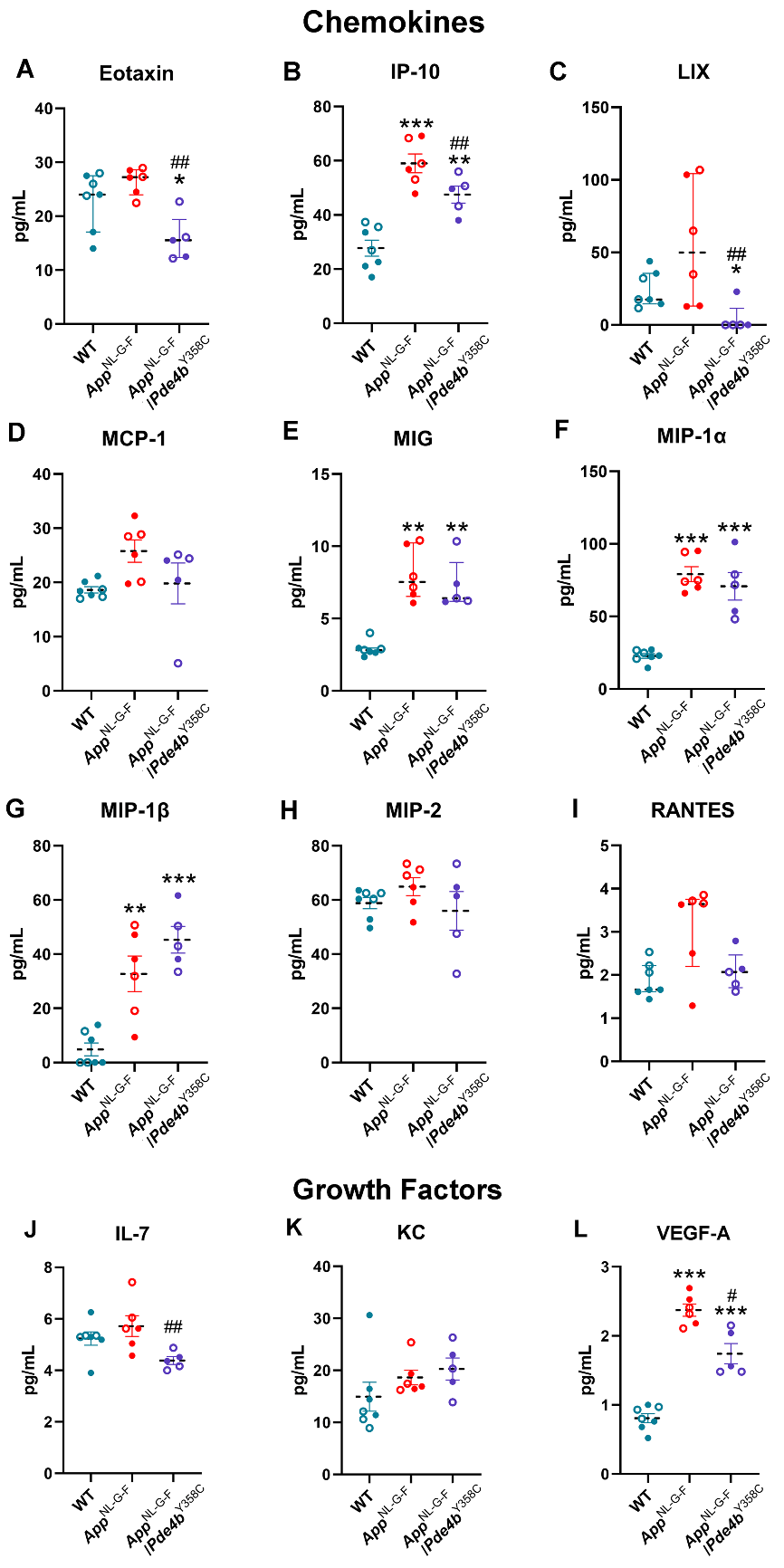


**Fig. S6. Chemokine and growth factor marker levels in brains from 12-month-old WT (*n* = 7), *App*^NL-G-F^ (*n* = 6) and *App*^NL-G-F^/*Pde4b*^Y358C^ (*n* = 5) mice.** **p* < 0.05; ***p* < 0.01; ****p* < 0.001 vs. WT. ^#^*p* < 0.05; ^##^*p* < 0.01 vs. *App*^NL-G-F^. Open circles, females; closed circles, males.


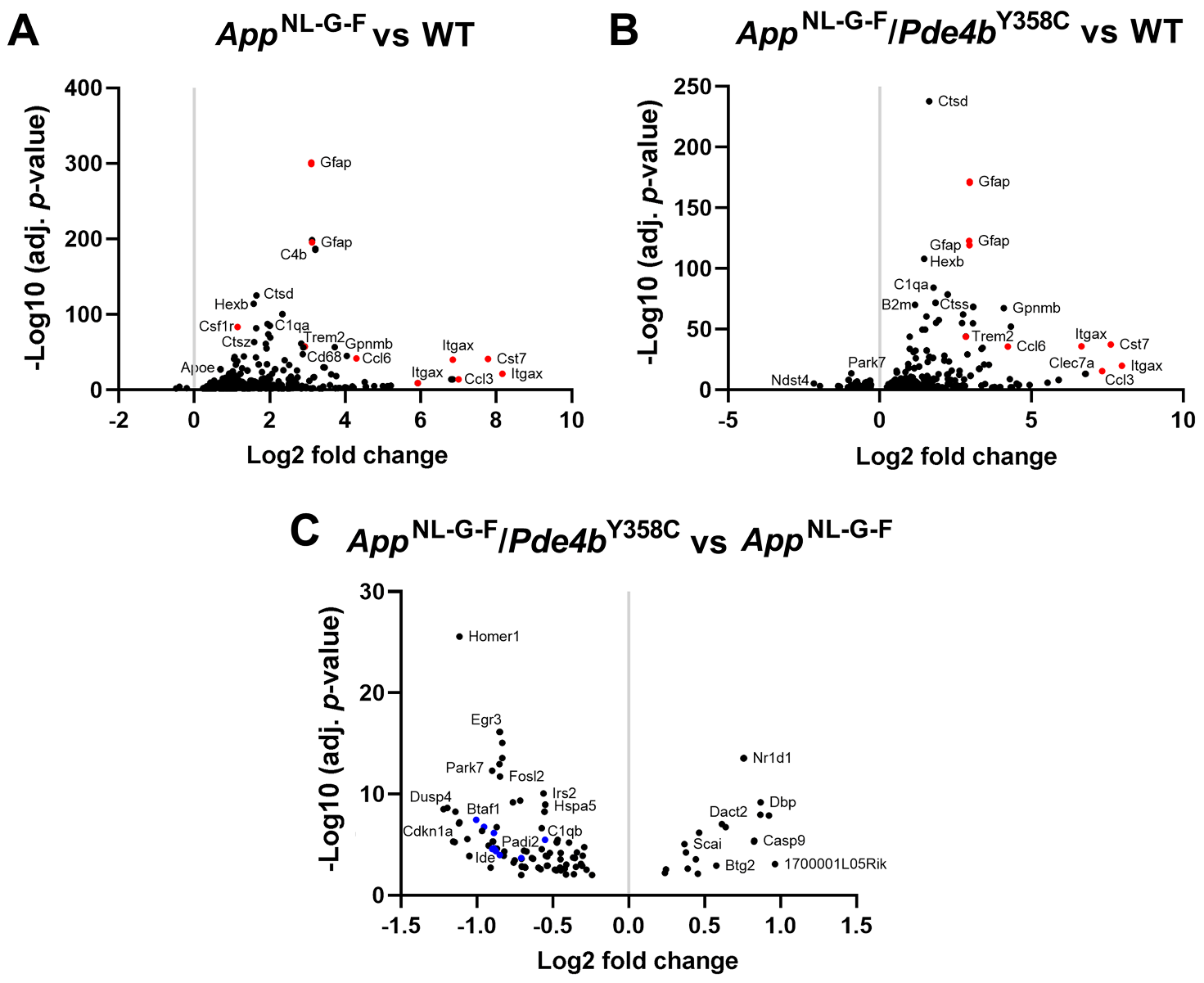


**Fig. S7. Differentially expressed gene transcripts in cerebral cortex from 12-month-old *App*^NL-G-F^ (*n* = 6) and *App*^NL-G-F^/*Pde4b*^Y358C^ (*n* = 5) compared with WT (*n* = 7) mice.** **A** Volcano plot displaying 531 DE transcripts in *App*^NL-G-F^ versus WT mice. **B** Volcano plot displaying 462 DE transcripts in *App*^NL-G-F^/*Pde4b*^Y358C^ versus WT mice. Red dots represent select transcripts encoding proteins implicated in neuroinflammation. **C** Volcano plot displaying 117 DE transcripts in *App*^NL-G-F^/*Pde4b*^Y358C^ versus *App*^NL-G-F^ mice. Blue dots represent 13 DE transcripts in *App*^NL-G-F^ versus WT mice most modulated by the PDE4B inhibition in *App*^NL-G-F^/*Pde4b*^Y358C^ mice. The vertical axis (*y*-axis) plots the -log_10_ adj. *p*-value and the horizontal axis (*x*-axis) displays the log_2_ fold change. Positive log_2_ fold change indicates up-regulated and negative log_2_ fold change indicates down-regulated expression compared with WT (A, B) or *App*^NL-G-F^ (C) mice.


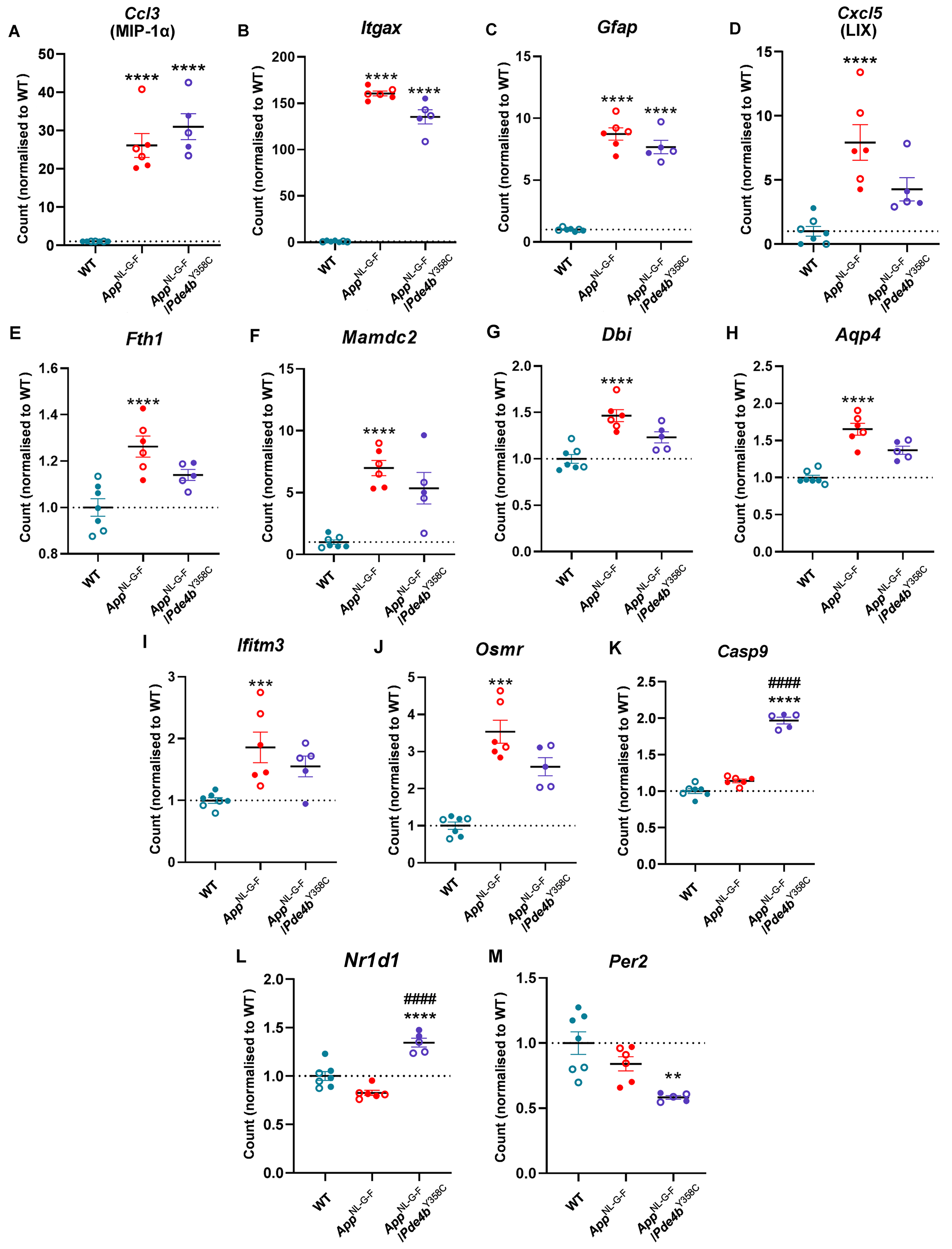


**Fig. S8. Exemplar differentially expressed gene transcripts in cerebral cortex from 12-month-old WT (*n* = 7), *App*^NL-G-F^ (*n* = 6) and *App*^NL-G-F^/*Pde4b*^Y358C^ (*n* = 5) mice.** **A** *Ccl3* (1 transcript), encoding MIP-1α*.* **B** *Itgax* (mean of 4 transcripts). **C** *Gfap* (mean of 4 transcripts). **D** *Cxcl5* (1 transcript), encoding LIX. **E** *Fth1* (mean of 2 transcripts). **F** *Mamdc2* (mean of 4 transcripts). **G** *Dbi* (mean of 2 transcripts). **H** *Aqp4* (mean of 9 transcripts). **I** *Ifitm3* (1 transcript). **J** *Osmr* (mean of 6 transcripts). **K** *Casp9* (mean of 3 transcripts). **L** *Nr1d1* (mean of 3 transcripts). **M** *Per2* (mean of 4 transcripts). **adj. *p* < 0.01; ****adj. *p* < 0.0001 vs. WT. ^####^adj. *p* < 0.0001 vs. *App*^NL-G-F^. Open circles, females; closed circles, males.


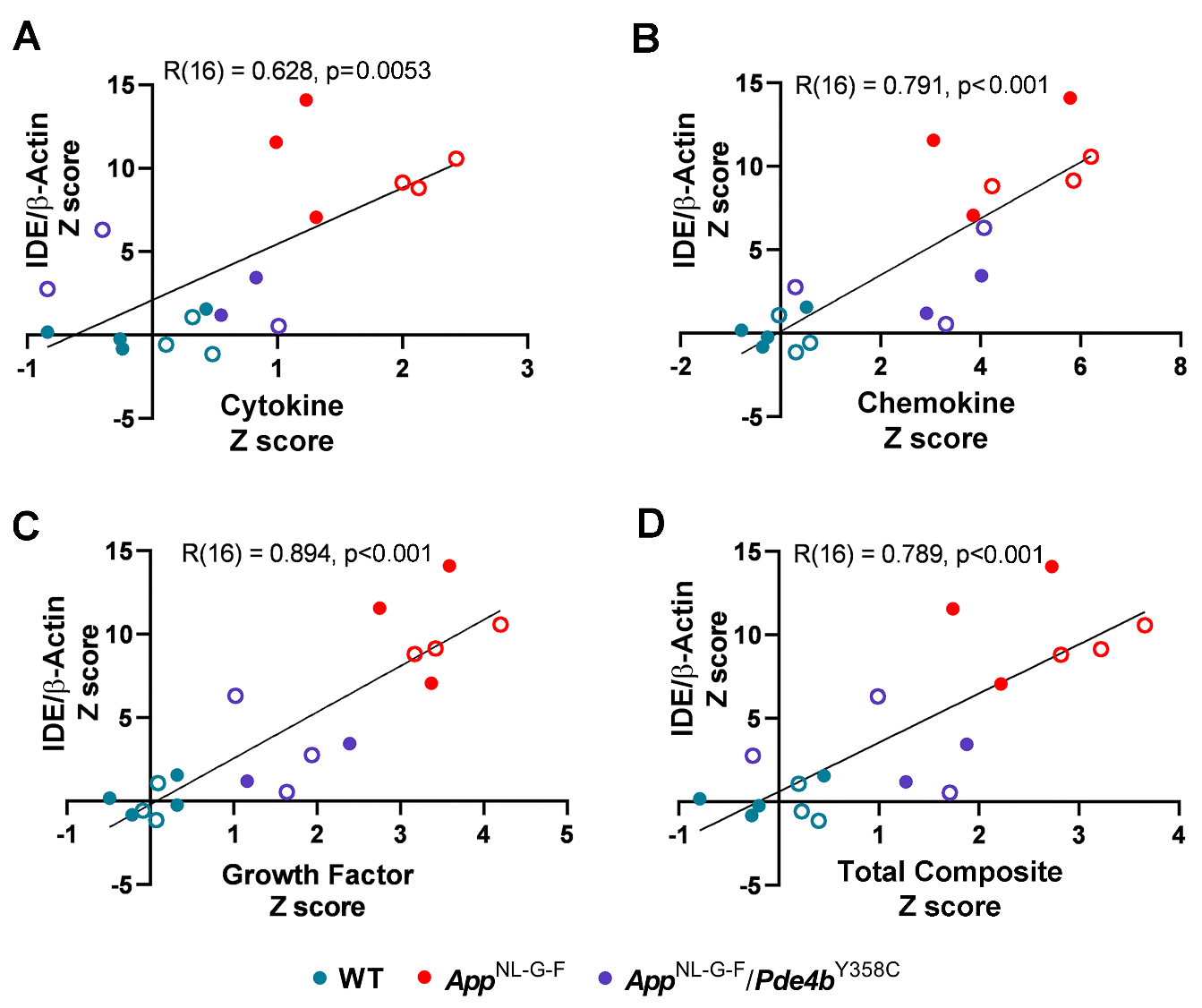


**Fig. S9. Correlations between IDE protein levels and inflammatory marker Z scores in brains from 12-month-old WT (*n* = 7), *App*^NL-G-F^ (*n* = 6) and *App*^NL-G-F^/*Pde4b*^Y358C^ (*n* = 5) mice.** **A** Correlation between IDE protein and cytokine Z score. **B** Correlation between IDE protein and chemokine Z score. **C** Correlation between IDE protein and growth factor Z score. **D** Correlation between IDE protein and total composite Z score. Open circles, females; closed circles, males.

**SUPPLEMENTARY REFERENCES**

1. Saito T, Matsuba Y, Mihira N, Takano J, Nilsson P, Itohara S, et al. Single App knock-in mouse models of Alzheimer's disease. Nat Neurosci. 2014;17:661–3.
2. McGirr A, Lipina TV, Mun HS, Georgiou J, Al-Amri AH, Ng E, et al. Specific Inhibition of Phosphodiesterase-4B Results in Anxiolysis and Facilitates Memory Acquisition. Neuropsychopharmacology. 2016;41:1080–92.
3. Al-Amri AH, Armstrong P, Amici M, Ligneul C, Rouse J, El-Asrag ME, et al. PDZD8 Disruption Causes Cognitive Impairment in Humans, Mice, and Fruit Flies. Biol Psychiatry. 2022;92:323–34.
4. Dawson N, Ferrington L, Lesch KP, Kelly PA. Cerebral metabolic responses to 5-HT2A/C receptor activation in mice with genetically modified serotonin transporter (SERT) expression. Eur Neuropsychopharmacol. 2011;21:117–28.
5. Hughes RB, Whittingham-Dowd J, Clapcote SJ, Broughton SJ, Dawson N. Altered medial prefrontal cortex and dorsal raphé activity predict genotype and correlate with abnormal learning behavior in a mouse model of autism-associated 2p16.3 deletion. Autism Res. 2022;15:614–27.
6. Franklin KBJ, Paxinos G. The Mouse Brain in Stereotaxic Coordinates. San Diego: Academic Press; 1997.
7. Allen Reference Atlas – Mouse Brain [brain atlas]. Available online at: atlas.brain-map.org.
8. Andrews S. FastQC: a quality control tool for high throughput sequence data. 2010. Available online at: http://www.bioinformatics.babraham.ac.uk/projects/fastqc.
9. Martin M. Cutadapt Removes Adapter Sequences from High-Throughput Sequencing Reads. EMBnet Journal. 2011;17:10–2.
10. Kuhn RM, Haussler D, Kent WJ. The UCSC genome browser and associated tools. Brief Bioinform. 2013;14:144–61.
11. Dobin A, Davis CA, Schlesinger F, Drenkow J, Zaleski C, Jha S, Batut P, Chaisson M, Gingeras TR. STAR: ultrafast universal RNA-seq aligner. Bioinformatics. 2013;29:15–21.
12. Nassar LR, Barber GP, Benet-Pagès A, Casper J, Clawson H, Diekhans M, Fischer C, Gonzalez JN, Hinrichs AS, Lee BT, Lee CM, Muthuraman P, Nguy B, Pereira T, Nejad P, Perez G, Raney BJ, Schmelter D, Speir ML, Wick BD, Zweig AS, Haussler D, Kuhn RM, Haeussler M, Kent WJ. The UCSC Genome Browser database: 2023 update. Nucleic Acids Res. 2023;51:D1188–95.
13. Okonechnikov K, Conesa A, García-Alcalde F. Qualimap 2: advanced multi-sample quality control for high-throughput sequencing data. Bioinformatics. 2016;32:292–4.
14. Wysoker A, Tibbetts K, Fennell T. Picard Tools Version 1.90. 2013. Available online at: http://picard.sourceforge.net.
15. Li H, Handsaker B, Wysoker A, Fennell T, Ruan J, Homer N, Marth G, Abecasis G, Durbin R; 1000 Genome Project Data Processing Subgroup. The Sequence Alignment/Map format and SAMtools. Bioinformatics. 2009;25:2078–9.
16. Thorvaldsdóttir H, Robinson JT, Mesirov JP. Integrative Genomics Viewer (IGV): high-performance genomics data visualization and exploration. Brief Bioinform. 2013;14:178–92.
17. Liao Y, Smyth GK, Shi W. The R package Rsubread is easier, faster, cheaper and better for alignment and quantification of RNA sequencing reads. Nucleic Acids Res. 2019;47:e47.
18. Love MI, Huber W, Anders S. Moderated estimation of fold change and dispersion for RNA-seq data with DESeq2. Genome Biol. 2014;15:550.
19. Benjamini Y, Hochberg Y. Controlling the false discovery rate: a practical and powerful approach to multiple testing. JR Statist Soc B. 1995;57:289–300.
20. Wu T, Hu E, Xu S, Chen M, Guo P, Dai Z, Feng T, Zhou L, Tang W, Zhan L, Fu X, Liu S, Bo X, Yu G. clusterProfiler 4.0: A universal enrichment tool for interpreting omics data. Innovation (Camb). 2021;2:100141.
21. Schneider CA, Rasband WS, Eliceiri KW. NIH Image to ImageJ: 25 years of image analysis. Nat Methods. 2012;9:671–5.
22. UniProt Consortium. UniProt: the Universal Protein Knowledgebase in 2023. Nucleic Acids Res. 2023;51:D523–31.
23. Cell Types Database: RNA-Seq Data – Mouse Whole Cortex and Hippocampus 10x. Available online at: https://portal.brain-map.org/atlases-and-data/rnaseq/mouse-whole-cortex-and-hippocampus-10x.
24. Yao Z, van Velthoven CTJ, Nguyen TN, Goldy J, Sedeno-Cortes AE, Baftizadeh F, et al. A taxonomy of transcriptomic cell types across the isocortex and hippocampal formation. Cell. 2021;184:3222–41.e26.
25. Cell Types Database: RNA-Seq Data – Human M1 10x. Available online at: https://portal.brain-map.org/atlases-and-data/rnaseq/human-m1-10x.
26. Bakken TE, Jorstad NL, Hu Q, Lake BB, Tian W, Kalmbach BE, et al. Comparative cellular analysis of motor cortex in human, marmoset and mouse. Nature. 2021;598:111–9.

**SUPPLEMENTARY FIGURE LEGENDS**

**Table S1.** Cerebral glucose utilization rates in WT, *App*^NL-G-F^ and *App*^NL-G-F^/*Pde4b*^Y358C^ mice

**Table S3.** Differentially expressed transcripts in *App*^NL-G-F^ v WT mice

**Table S4.** Differentially expressed transcripts in *App*^NL-G-F^/*Pde4b*^Y358C^ v WT mice

**Table S5.** Differentially expressed transcripts in *App*^NL-G-F^/*Pde4b*^Y358C^ v *App*^NL-G-F^ mice

**Table S6.** Differentially expressed transcripts in *App*^NL-G-F^ v WT mice unaffected by PDE4B inhibition

**Table S7.** Differentially expressed transcripts in *App*^NL-G-F^ v WT mice modulated by PDE4B inhibition

**Table S9.** Differentially expressed transcripts in *App*^NL-G-F^/*Pde4b*^Y358C^ v WT driven by PDE4B inhibition

**Table S10.** PDE4 transcripts identified in cerebral cortical samples from 12-month-old *App*^NL-G-F^, *App*^NL-G-F^/*Pde4b*^Y358C^ and WT mice
